# Multiplex imaging of murine bone marrow using Phenocycler 2.0™

**DOI:** 10.1038/s41375-025-02596-5

**Published:** 2025-04-11

**Authors:** Sonali J. Karnik, Connor Gulbronson, Paige C. Jordan, Rahul Kanumuri, Baskar Ramdas, Ramesh Kumar, Melissa L. Hartman, Izza Khurram, Drew M. Brown, Karen E. Pollok, Pratibha Singh, Reuben Kapur, Melissa A. Kacena

**Affiliations:** 1 Department of Orthopaedic Surgery, Indiana University School of Medicine, Indianapolis, IN USA; 2 Indiana University Cooperative Center of Excellence in Hematology (CCEH), Indianapolis, IN USA; 3 Indiana Center for Musculoskeletal Health, Indiana University School of Medicine, Indianapolis, IN USA; 4 Division of Nephrology, Department of Medicine, Indiana University, Indianapolis, IN USA; 5 Weldon School of Biomedical Engineering, Purdue University, Indianapolis, IN USA; 6 Department of Pediatrics, Herman B Wells Center for Pediatric Research, Indiana University School of Medicine, Indianapolis, IN USA; 7 Department of Anatomy and Cell Biology, Indiana University School of Medicine, Indianapolis, IN USA; 8 Richard L. Roudebush VA Medical Center, Indianapolis, IN USA

**Keywords:** Haematopoietic stem cells, Haematopoiesis

## Abstract

Bone marrow (BM) is a tissue that is of great importance to several areas of basic and translational research, including hematology, oncology, bone biology, and immunology. It is unique in that it is gelatinous in nature but housed in a hard casing of bone. Traditionally, flow cytometry and immunofluorescence (IF) techniques have been employed to study the composition of cellular interactions and elements of the BM. However, it has been challenging to study the BM in an unperturbed state using multiple fluorescent probes at a time to fully appreciate the diverse cell populations and their interactions and relative positioning with each other. This protocol addresses how Phenocycler 2.0^TM^, which uses co-detection by indexing (CODEX) in conjunction with HALO 4.0^TM^ image analysis software, can overcome the obstacles faced by traditional techniques used to study the BM in an unperturbed state.

## Introduction

Bone marrow (BM) is the primary site of hematopoiesis in mammals and, therefore, is of great importance in the fields of non-malignant and malignant hematology [1, 2]. Different cells such as hematopoietic stem cells (HSCs), common myeloid progenitors (CMPs), common lymphoid progenitors (CLPs), macrophages, megakaryocytes (MKs), T cells, B cells, mast cells, endothelial cells (ECs), and others reside in the BM and interact with each other [3–10]. These interactions are based on molecular signals as well as spatial relationships that cells have with each other [11, 12]. Researchers currently employ established techniques such as flow cytometry and immunofluorescence (IF) to study the BM [13]. These techniques are useful and have been utilized to study the composition of BM tissue; however, they have drawbacks [13, 14]. For example, flow cytometry uses dissociated BM to quantify the cells. Due to the dissociation of the tissue, the spatial and structural information is lost. With IF, there is a limit on the number of cell markers you can include before it becomes impossible to separate the individual signal spectrally. Typically, eight markers are viewed as the upper end of spectral unmixing. Therefore, visualizing unperturbed BM with its cellular and structural features intact, without the limitations of the number of cell markers, can benefit hematology research by making it possible to interrogate a single section for more than eight cell markers at a time. This becomes especially beneficial in studying rare cell types such as hematopoietic stem and progenitor cells (HSPCs), which are identified by staining cells with antibodies against 5–7 different markers [15–18]. An additional benefit of using a multiplex imaging platform is that different cellular and structural niches can be studied for spatial context. BM niches and their vicinity to vasculature can give researchers an idea of how cellular and structural components of the BM interact with each other.

The Phenocycler 2.0^TM^ is available commercially from Akoya Biosciences® and achieves multiplexing by co-detection by indexing (CODEX) as a multiplex imaging technique. Multiplex imaging of murine non-hematopoietic niche was demonstrated by Coutu et al. in 2017, which used multicolor three-dimensional imaging of murine femurs to map the non-hematopoietic cells and other structural components of the BM [19]. Some multiplex imaging techniques have been developed to study different aspects of BM functions, such as myelopoiesis. One such technique developed by Zhang et al. in 2021 used a combination of inducible Cre mice expressing fluorescence for specific lineage markers (confetti mice), confocal imaging, and sequential building of the map of the fluorescent cells of the myeloid lineage in the murine BM [20]. Recently, Bandyopadhyay et al. demonstrated that CODEX can be used to image BM from human samples obtained from orthopedic hip replacement surgery [21]. This study showed that CODEX can be a useful tool to study and build an atlas encompassing the major cell types in healthy human and acute myeloid leukemia samples.

Murine models are extensively used in pre-clinical research and are an important tool for understanding the pathways and mechanisms of different disease states. By manipulating specific genes in mice, one can assess how genes and/or proteins impact the development and localization of specific cells within the BM cavity with respect to other cells. Here, we show the use of the Phenocycler 2.0^TM^ multiplex imaging platform adapted for cryosectioned murine BM tissue and cell type identification by using HALO 4.0™ (henceforth referred to as HALO™) image analysis software. Our objective was to develop a protocol for processing murine BM and adapting the Phenocycler 2.0™ for imaging cell surface proteins on HSPCs and more committed cells, as well as structural markers that constitute the murine BM microenvironment.

## Phenocycler 2.0^TM^ multiplex imaging

The Phenocycler 2.0^TM^ is a multiplex imaging platform developed by Akoya Biosciences® that utilizes a technology called CODEX [22, 23]. CODEX requires the construction of an antibody panel where each antibody has been conjugated with a unique DNA oligo tag referred to as oligo-barcodes. This panel is then applied simultaneously during staining to a tissue of interest. During an imaging experiment, three complementary barcodes are added, which then bind and ‘reveal’ the antibody. After each addition, the oligo-barcodes are removed, and a new set is added and imaged [22, 23]. This process is automated, with images taken in three different channels (488 or 750, 550, and 647 nm). The resulting images are stitched together by the system’s software, creating a final image (.qptiff) that displays the different markers and their location in the tissue.

**Fig. 1:**
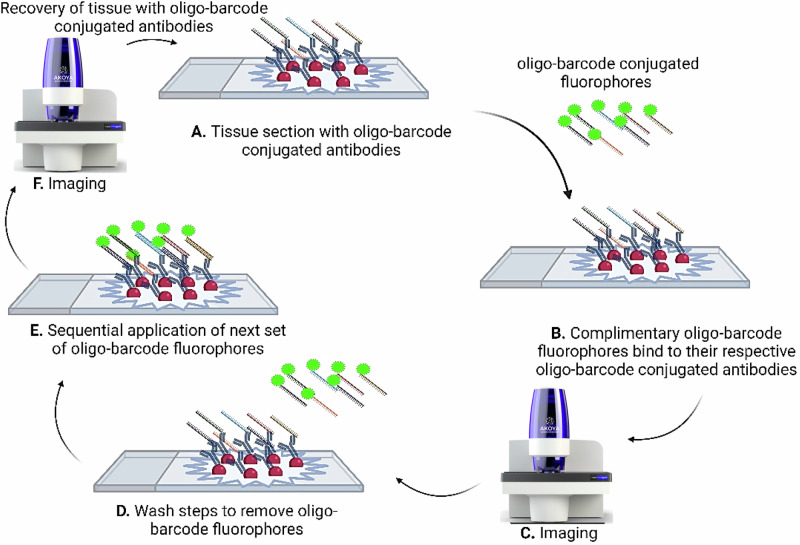
Phenocycler 2.0^TM^ multiplex imaging schematic showing the sequential steps of oligo-barcode addition, imaging, and washing out the oligo-barcodes. The process follows these steps: **A** Addition of oligo-barcode conjugated antibodies on tissue section on a glass slide. **B** Complimentary oligo-barcode fluorophores bind to their respective barcode conjugated antibodies. **C** Imaging. **D** Wash steps to remove oligo-barcode fluorophores. **E** Sequential application of next set of oligo-barcode fluorophores. **F** Imaging.

Figure 1 illustrates the steps involved in the process of Phenocycler 2.0^TM^ multiplex imaging. One notable advantage of the Phenocycler 2.0^TM^ is its capability to re-run the sample tissue post-run, eliminating the need to stain another tissue section, and potentially reducing the usage of antibodies, as well as saving time and resources. Additionally, the system offers the flexibility to use traditional non-fluorescent stains such as Hematoxylin and Eosin (H&E) to stain the same tissue section post-run.

## Challenges involved in imaging BM and potential resolution

Imaging BM can be difficult due to the inherent nature of the tissue. The BM is gel-like and surrounded by hard, mineralized bone. Additionally, BM is highly vascular, with capillaries and blood vessels running through it. Processing the BM for imaging can damage its structure and blood vessels. The tissue’s inherent autofluorescence can also make it challenging to image certain cell markers that are found in rare cell populations. Choosing the right method for fixing, embedding, and sectioning the bone can improve the imaging process. In our protocol, we compared various commonly used fixatives for murine femurs for cryosectioning. Unlike the wide range of pre-conjugated antibodies available for human tissues, Akoya Biosciences® does not offer pre-conjugated antibodies for paraffin-embedded murine tissue. To include more cell or structural markers for imaging murine BM, custom-conjugated antibodies were created as described in this protocol. Paraffin embedding may require antigen retrieval, which can destroy delicate epitopes and distort tissue morphology [24–27]. Given these limitations, cryosectioning was a more suitable option for identifying the markers examined in our panel for the BM. We found that perfusion with 1× PBS, a common practice for removing red blood cells from tissues, disrupts BM vasculature (Supplemental Fig. 1). Optimizing the conditions for fixing and embedding bone is crucial for obtaining good quality cryosections and reducing interference due to autofluorescence. By reducing the number of processing steps, we were able to maintain the tissue’s structural integrity, which is important for understanding cell locations and vasculature.

## Rationale for improved imaging and image processing pipeline for Phenocycler 2.0™ BM imaging

The Phenocycler 2.0™ comes with built-in image processing, and the resulting image can be viewed in Phenochart™ software (Akoya Biosciences®). Some of the cell and structural markers in our panel produce a dim signal. Despite selecting the best possible tissue processing conditions, the specific signal for some stem cell markers, such as CD117, remained dim, which is further complicated by the relative rarity of CD117 marker in wild-type (WT) tissues, causing it to be difficult to visualize using the manufacturer provided image processing and visualization software (Supplemental Fig. 2). Along with CD117, there were other markers such as CD41, α-SMA, CD48, Endomucin, and many of the extracellular matrix (ECM) markers that could not be visualized accurately using the manufacturer’s recommended image processing pipeline and Phenochart™ (Supplemental Fig. 2A). Manufacturer built-in image processing utilizes two blank cycles (first and last cycle of the run) for subtracting background set to a singular exposure time (150 ms as default). The exposure times for these blanks can be changed; however, they cannot be set at different exposure times for each individual marker. Due to the variety of biological markers, the abundance of antigens, and the quality of antibodies among the antibody panel, optimization of exposure times is critical. For example, our antibody panel contains markers that need higher exposure times for optimal visualization, such as Endomucin (600 ms), CD31 (800 ms), CD115 (800 ms), and other structural markers (Supplemental Fig. 3). The panel also includes markers that need a shorter exposure time, such as CD41 (75 ms) (Supplemental Fig. 2A). This range of exposure times poses a challenge for picking a singular exposure time suitable for background subtraction. With a low exposure blank, there is insufficient removal of background in high-exposure marker channels. With a high-exposure blank, there is an over-compensation, and we risk losing a genuine signal to this post-processing step. To overcome these obstacles for obtaining good quality multiplex images for murine BM that are also accurate, we optimized and profoundly improved the existing manufacturer’s protocol. Interleaved blanks with exposure times similar to the corresponding marker, as well as an initial group of blanks (called “pre-treat”) were added. The images were processed from ‘.raw.qptiff’ files generated from the Akoya Biosciences® built-in image processing as explained in detail in the section on multiplex imaging and background subtraction below. The rationale for this “pre-treat” blanking scheme was to mitigate autofluorescence build-up and to increase the accuracy of background subtraction for each marker individually to obtain a better signal-to-noise ratio. This was needed to identify rare cell markers (such as CD117), which also were low in signal intensity. Accurate visualization is important for accurate interpretation of results, especially when Phenocycler 2.0™ multiplex imaging is used in studies of diseased states of BM and the effectiveness of treatments.

## Identification of cell markers for imaging the BM

A crucial step in imaging the BM is building a robust panel of antibodies and reporters with their corresponding oligo-barcodes. Three channels are available for each antibody: Atto550, AlexaFluor™ 647, and AlexaFluor™ 750. Choosing which antibody to place in which channel is an important step based on the abundance of the cell marker and the sensitivity of the channel to the camera. We avoided using 488 nm due to high autofluorescence from Red Blood Cells (RBCs) and other tissue components that exhibit strong autofluorescence (due to the porphyrin ring structures in the heme group) in the 488 nm channel [28]. Abundant cell markers were assigned to the AlexaFluor™ 750 channel as it is the lowest camera sensitivity channel. The least abundant cell markers were assigned to the Atto550 channel, which has the highest camera sensitivity of our three used channels. The cell markers expressed that were not rare but not too abundant were assigned to AlexaFluor™ 647 nm. The complete panel of cell markers and the cell types that we identified using these markers are listed in Table 1. Table 2 shows the channels assigned to antibodies, the cell markers, and the corresponding oligo-barcodes.

**Table 1:** Cell markers, cell types, and structures of BM.

Cell types or structures	Cell markers	Identification conditions
B Cells	CD45, B220	Positive
Macrophages	CD45, F4/80	Positive
Mast cells	Lineage*(B220, Gr1, Ter119)	Negative
	CD45, CD48 &/ CD117	Positive
Neutrophils	CD45, GR1	Positive
Erythroid cells	CD45, Ter119, CD71	Positive
	GR1, B220	Negative
MKs (polyploid nucleus)	CD45, CD41 &/ CD61, CD110	Positive
Primitive progenitor (LSK fraction)	Lineage*(B220, Gr1, Ter119)	Negative
	CD117, SCA1	Positive
Common myeloid progenitor (CMP)	Lineage*(B220, Gr1, Ter119) SCA1	Negative
	CD117	Positive
Common lymphoid progenitor (CLP)	Lineage*(B220, Gr1, Ter119) CD117	Negative
	SCA1	Positive
Osteoclasts (multinucleate cells)	CD45, CD115	Positive
Capillaries and sinusoids	CD31, SCA1, Endomucin	Positive
Nerves	Calcitonin gene-related peptide (CGRP)	Positive
Structural proteins	Fibromodulin, Fibulin2, Lumican, EGF Containing Fibulin Extracellular Matrix Protein 2 (EFEMP2), Emillin2, Cartilage Associated Protein (CRTAP), Pigment epithelium-derived factor (PEDF), Collagen Type I-a (Col1a), α smooth muscle actin (α- SMA)	Positive

Some cell types are identified as Lineage (B220, Gr1, Ter119) negative. These markers are indicated as "Lineage*" in bold letters. Based on other conditions needed to identify these cell types, other markers such as SCA1 or CD117 are also added in bold as per the case.

*LSK* Lineage-SCA1+ CD117+.

**Table 2:** Antibodies against the cell markers, channels, and oligo-barcode numbers (manufacturer-assigned).

Antibody	Channel	Conjugated (custom or purchased)	Dilution factor used for staining	Oligo-barcode	Manufacturer Pre-conjugated antibody / Oligo-barcode catalog number
CD31	550	Commercial	1–100	2	4250001
CD41	647	Custom	1–200	15	5550008
CD45	750	Commercial	1–100	7	4450002
CD61	647	Custom	1–100	42	5550015
CD71	647	Commercial	1–100	27	4550111
CD117	750	Custom	1–100	19	5450002
B220	750	Commercial	1–100	10	4450006
F4/80	647	Commercial	1–200	6	Leinco-F401
Gr1	647	Custom	1–400	3	5550017
Sca1	647	Custom	1–200	33	5550013
Ter119	550	Custom	1–100	5	5450024
CD48	750	Custom	1–100	22	5450003
CD110	750	Custom	1–100	468	**
CD115	550	Custom	1–100	500	**
Endomucin	550	Custom	1–100	41	5250008
Fibromodulin	550	Custom	1–100	40	5250017
Fibulin2	647	Custom	1–100	31	5550004
CGRP	550	Custom	1–100	518	**
EFEMP2	550	Custom	1–100	35	5250007
Lumican	750	Custom	1–100	34	5450007
CRTAP	647	Custom	1–100	43	5550005
Col1a	550	Custom	1–100	135	**
α-SMA	647	Custom	1–200	45	5550016
Emillin2	647	Custom	1–100	24	5550010
PEDF	647	Custom	1–100	36	5550014

** Catalog number not available. Purchased from the manufacturer as part of a special program. Contact the manufacturer.

## Materials

### Animals

- 12–14-week-old male C57BL/6J mice (In Vivo Therapeutics Core, Indiana University Simon Comprehensive Cancer Center).

C57BL/6J mice were housed in a pathogen-free facility at Indiana University School of Medicine, Indianapolis. All animal studies were conducted with approval from the Indiana University Laboratory Animal Resource Center.

### Reagents

- Methanol (ThermoFisher, cat #176840010)
- Acetone (ThermoFisher, cat # L10407)
- 16% w/v aqueous solution of Paraformaldehyde (Thermofisher, cat # 043368.9 M)
- 2× Laemmli sample buffer (Bio-Rad, cat# 1610737)
- Novex™ Tris-Glycine Mini Protein Gels, 4–20%, 1.0 mm, WedgeWell™ format (ThermoFisher, cat # XP04205BOX)
- Coomassie Brilliant Blue G 250 (Sigma, cat# 115444)
- Glacial acetic acid (ThermoFisher, cat# 9526-33)
- 10% Neutral Buffered Formalin (EKI, cat# 4499-GAL)
- EDTA (ThermoFisher, cat# 17892)
- Optimal Cutting Temperature (O. C. T.) Compound (Fisher Scientific, cat# 23-730-571)
- Sucrose (Fisher Scientific, cat# S5-3)
- Anti-mouse CD61(Biolegend-104325)
- Anti-mouse CD150 (Biolegend 115949)
- Anti-mouse Ly-6G/Ly-6C (Gr1) (Biolegend-108435)
- Anti-mouse Ter119 (Biolegend-116253)
- Anti-mouse CD41 (Biolegend-133939)
- Anti-mouse CD117(c-kit) (Biolegend-135114)
- Anti-mouse Sca1 (ThermoFisher 14-5981-82)
- Pierce Antibody Clean-up Kit (ThermoFisher 44600)
- Oligo-barcode and reporter information (refer to Table 2)
- Akoya pre-conjugated antibody anti-mouse CD31-BX002 (Akoya, cat# 4250001)
- Akoya pre-conjugated antibody anti-mouse CD45-BX007 (Akoya, cat# 4450002)
- Akoya pre-conjugated antibody anti-mouse CD45R/B220-BX010 (Akoya, cat# 4450006)
- Akoya pre-conjugated antibody anti-mouse CD71-BX027 (Akoya, cat# 4550111)
- Akoya antibody conjugation kit (Akoya, cat# 7000009)
- Akoya staining kit (Akoya, cat# 7000008)
- Akoya 96-well plates for Phenocycler (Akoya, cat# 7000006)
- Akoya 96-well plate seals for Phenocycler (Akoya, cat# 7000007)
- Corning Cell-Tak™ (Corning CLS354240)
- ProLong Diamond antifade mountant with DAPI (Invitrogen, cat# P36962)
- Rabbit F (ab’)2 Anti-mouse FITC IgG (H+L) secondary antibody (Southern Biotech cat# 6120-02)
- Rabbit F (ab’)2 Anti-rat FITC IgG (H+L) secondary antibody (Southern Biotech cat# 6130-02)
- Rabbit F (ab’)2 Anti-goat FITC IgG (H+L) secondary antibody (Southern Biotech cat# 6020-02)
- Bovine Serum Albumin (Sigma cat# A3733)

### Equipment

- BZ-X810 fluorescent microscope (Keyence, Itasca, Illinois)
- Gel Electrophoresis (Bio-Rad ChemiDoc MP Imaging System, Hercules, California)
- Phenocycler 2.0™ (Akoya, Marlborough, Massachussetts)

### Software programs

- HALO 4.0^TM^ (Indica Labs, Albuquerque, New Mexico)

### Procedure

After the channels and oligo-barcodes were assigned to the cell markers, we next custom-conjugated the cell markers that were not available commercially to their respective oligo-barcodes.

The process described below is adapted from [23]:

Custom conjugating antibodies to Phenocycler Fusion™ oligo-barcodes. The manufacturer uses Phenocycler Fusion™ to refer to the reagents used for imaging assays and associated processes for Phenocycler Fusion™ microscope. The automated platform, including the microscope, fluidics, software to run the imaging assay, and image output, is collectively referred to as Phenocycler 2.0™ by the manufacturer.

The protocol to custom conjugate the antibodies to the oligo-barcodes was obtained from Akoya, and the manufacturer’s instructions were followed. The steps are as follows:

1. Purified stock solution of antibodies in 1× PBS was prepared. Antibodies were free of carrier proteins and sodium azide. If antibodies contained carrier proteins and sodium azide, they were purified using a protein purification kit.
2. Volume of the solution corresponding to 50 μg of antibody was calculated.
3. The following reagents were retrieved at the start of the process:
  Reduction Solutions 1 & 2
  Filter Blocking Solution

4. The following reagents were retrieved in ~1 h after starting the process:
  Conjugation Solution
  Barcodes

5. The following reagents were retrieved in ~3 h:
  Purification Solution
  Antibody Storage Solution
  Purified Antibody

a. 50 kDa MWCO filter was labeled for each antibody.

c. 500 μl of Filter Blocking Solution was added to the top of each 50 kDa MWCO filter. The filters with their collection tubes were then centrifuged at 12,000 × *g* for 2 min.

e. All the liquid that was left was removed and discarded.

f. 50 μg of the purified antibody in a volume of 100 μl or greater was added to the filters and collection tubes and then centrifuged 12,000 × *g* for 8 min. Flow-through was discarded.

g. Antibody Reduction Master Mix was prepared based on the number of Phenocycler Fusion™ antibody conjugates as shown below in Table 3.

**Table 3:** Preparation of reduction master mix for antibody conjugation.

No. of antibodies	1	2	3	4	5	6	7	8
Reduction solution 1 [µL]	6.6	13.2	19.8	26.4	33	39.6	46.2	52.8
Reduction solution 2 [µL]	275	550	825	1100	1375	1650	1925	2200
Total [µL]	281.6	563.2	844.8	1126.4	1378	1689.6	1971.2	2252.8

We next verified if the conjugation of the antibodies to their respective oligo-barcodes was successful and if the conjugated antibodies remained functional after the chemical modification during the conjugation steps. Verification steps included gel electrophoresis and immunofluorescence validation to not only confirm the success of the conjugation to the barcodes but also to verify if the binding sites for the antibodies are not blocked or rendered unusable for imaging.

### Gel electrophoresis

Protein gel electrophoresis was performed to verify the success of antibody conjugation for any antibodies not obtained from Akoya Biosciences®. The detailed procedure is as follows:

1. 5 μl of each conjugated antibody and 2 μl of unconjugated antibody (used as a control) were diluted to a final volume of 10 μl and mixed with 10 μl of 2× Laemmli sample buffer from Bio-Rad.
2. The samples were then denatured at 95 °C in a dry bath for 10 min. Subsequently, each sample was loaded into the wells of a 10-well Novex WedgeWell 4–20% Tris-Glycine gel, and the gel was electrophoresed at 100 V for 1 h until the process was complete.
3. After the gel run, the gels were gently removed from the cassette and rinsed once with distilled water.
4. The gel was then stained for one hour using a Coomassie Brilliant Blue staining solution (comprising 0.1% Brilliant Blue G from Sigma, 50% methanol, and 10% glacial acetic acid).
5. Subsequently, the gel was destained using a destaining buffer (consisting of 50% methanol and 10% glacial acetic acid) until complete destaining was achieved.
6. Images were captured using the ChemiDoc MP Imaging System by Bio-Rad.

As shown in Supplemental Fig. 4, the unconjugated antibody showed one band for the light chain of the antibody and one for the heavy chain of the antibody, whereas the conjugated antibody showed a shift due to a higher band size due to the addition of an oligo-barcode. Multiple bands are frequently observed on the conjugated antibody, suggesting multiple oligos have been conjugated. To ensure that the antibody remains functional after conjugation, tissue is prepared and IF validation performed as described below.

### Selection of the best fixative for Phenocycler 2.0^TM^ imaging of murine BM

Different fixatives can be used to fix bones, depending on the area and the desired imaging feature. 10% Neutral Buffered Formalin (NBF) is a commonly used fixative that has been used to fix bones such as femurs, tibiae, ulnae, radii, cranium, sternum, and vertebral column. However, due to the nature of formalin (37% formaldehyde with 6–12% methanol), formalin containing fixative can result in autofluorescence, which can hamper signal recognition in sensitive imaging such as with the Phenocycler 2.0™.

A pilot study was conducted to see which method of fixation provided the best results with the least autofluorescence while preserving the tissue architecture and internal features of interest utilizing Phenocycler imaging. The conditions and details for fixation, decalcification, embedding, and sectioning are provided in Table 4. All the femurs treated with the indicated fixatives were decalcified in 10% EDTA on a shaking platform for approximately 2 weeks post-fixation. The completion of decalcification was verified by x-ray imaging of the bones. Decalcification was considered complete if the x-ray was transparent, indicating the removal of mineral content. The femurs were washed in 1× PBS and then put in 30% sucrose solution overnight at 4 °C before embedding in O.C.T. compound.

**Table 4:** Conditions and details for fixing femurs for cryosectioning.

Fixative:	4% Paraformaldehyde (PFA)	10% NBF	Methanol: Acetone (1:1)
**Duration of fix:**	Overnight	48 h	30 min
**Decalcification:**	EDTA (~2.5 wks)	EDTA (~2.5 wks)	EDTA (~2.5 wks)
**Sample size:**	3	3	3
**30% Sucrose at 4 °C**	Overnight	Overnight	Overnight
**Embedding:**	O. C. T.	O. C. T.	O. C. T.
**Section thickness (µm):**	10	10	10

*O.C.T.* optimal cutting temperature, *NBF* neutral buffered formalin.

The metric used to assess the quality of the fixation included identifying the best fixative for bone cryosections that not only preserves the architecture and cell niches in the marrow but also generates little to no autofluorescence, To address this aspect of tissue processing, we imaged the tissue sections on a Phenocycler 2.0^TM^ system without any antibodies before IF validation of the conjugated antibodies to test which condition would work the best with respect to autofluorescence for BM imaging. This was achieved by testing a series of fixative conditions with mock runs on the Phenocycler 2.0^TM^ system. The results suggest that Methanol: Acetone (1: 1) fixative resulted in the least amount of background autofluorescence from processing the tissue and the quality of cryosections. Representative images are shown in Supplemental Fig. 5.

### Preparation of femurs for cryosectioning

After selecting the appropriate method of fixation and processing, the femurs were embedded in O. C. T. compound for cryosectioning. The detailed steps from isolating femurs from mice to embedding and cryosectioning are described below:

1. Femurs were Isolated from 12–15-week-old male C57BL/6J mice. Soft tissue was removed from the femurs. Femurs were placed in 1:1 Methanol: Acetone fixative (kept cold at −20 °C) and fixed for 30 min at −20 °C.
2. After fixation, femurs were washed in 1× PBS to rid the tissue of any fixative solution.
3. Next, femurs were placed in 10% EDTA decalcification solution on a rocking platform for ~2–2.5 weeks.
4. Complete decalcification was verified by x-ray imaging. Complete decalcification is required to ensure proper sectioning of the tissue. Incomplete decalcification may lead to poor, ruptured sections affecting BM continuity.
5. After decalcification, femurs were washed in 1× PBS.
6. Femurs were then placed in 30% sucrose solution overnight at 4 °C.
7. Next day, femurs were embedded in O. C. T. in cryomolds, and the blocks were stored at −80 °C until sectioning.
8. Femurs were sectioned on Cell-Tak™ coated or Silane-treated slides at 10 µm thickness, ensuring the section lies flat on the slide without any folds or creases. Folds and creases in the section can result in tissue lifting off the slide during Phenocycler runs.
9. Slides containing Cryosections were stored at −80 °C.

### Immunofluorescent validation

For the IF validation of custom-conjugated antibodies, based on the results from the preliminary Phenocycler run (as discussed above), Methanol: Acetone fixed cryosections of EDTA decalcified femurs of the C57BL/6J mice were used. The detailed procedure is described below:

1. The slides with cryosectioned tissues were equilibrated in a humidity chamber for 10 min at room temperature. For all the following steps until mounting (steps 2–6), the tissue slides were kept in a humidity chamber to prevent the tissue from drying.
2. Blocking buffer (1% bovine serum albumin in 1× PBS) was added to the tissues for 30 min at room temperature.
3. Primary antibodies diluted to the appropriate dilution factor in blocking buffer were added to the tissues and kept overnight at 4 °C (Table 2).
4. The tissues were washed via gentle pipetting with 1× PBS to remove any unbound primary antibody. Caution: Tissue can be easily damaged during washing steps and is susceptible to drying.
5. Appropriate secondary antibodies, diluted in the blocking buffer (1:500), were added to the tissues and kept for 30 min at room temperature in the dark.
6. The tissues were washed in 1× PBS twice for 10 min at room temperature in the dark. This was done gently to remove any unbound secondary antibodies.
7. After the washes, an antifade mounting medium with DAPI was used to stain the nuclei and to protect the tissues from fading during fluorescence imaging.
8. After curing for 24 h, fluorescence images were captured using a Keyence BZ-X810 fluorescent microscope.

After verifying the conjugation of oligo-barcodes to antibodies and IF validations (Supplemental Fig. 6), the next step was the selection of the proper tissue processing for the BM. We tested perfusion, which is a common technique used to reduce autofluorescence from RBCs in the BM discussed in detail in the next section.

### Perfusion vs non-perfusion approach to deplete RBCs from the BM cryosection

Autofluorescence from RBCs is a significant concern when it comes to imaging tissues that are rich in vasculature and have abundant RBCs. BM, being the site of hematopoiesis and being a vascular tissue, has abundant RBCs. Even though we selected channels that give the least autofluorescence from RBCs (550, 647, and 750 nm), RBCs still show some weak autofluorescence in all channels (Supplemental Fig. 1A–C). Indeed, as shown in Supplemental Fig. 1A–C, RBCs might interfere with image analysis by fluorescing in all the channels. This dilutes the specific signal from the actual cell markers, making it difficult to interpret the data.

One commonly used method to reduce the number of RBCs in tissues is to perfuse the animal with 1× PBS, followed by a fixative such as 4% PFA to internally fix tissues. However, for Phenocycler imaging of the BM, we used a different fixative (described above); therefore, the mice were perfused only with 1× PBS. It is important to note that using Methanol, especially in the presence of water or a water-rich environment such as biological tissues, is exothermic, leading to excessive heat generation and potential tissue degradation.

Different speeds and durations of perfusion were tested to preserve BM architecture. However, even at reduced speed, perfusion damaged the marrow and vasculature, resulting in poor imaging quality for studying microarchitecture and vasculature. As shown in Supplemental Fig. 1D, E, we observed that perfusion, even at a reduced speed, destroys the marrow architecture and vasculature and, in turn, is not effective in getting rid of the RBCs to an extent to which they do not interfere with the specific cell marker signal. Importantly, perfused BM led to wide gaps in the marrow with a concomitant loss of vascular structures such as capillaries that can be seen in non-perfused BM. Therefore, our results suggest that the use of perfusion is likely to be associated with poor multiplex imaging quality of the BM, particularly as it relates to studying the microarchitecture and vasculature.

### The workflow involving the selection of cell markers and extending to cryosectioning

A schematic of the workflow from designing and planning to tissue preparation for staining is described in Supplemental Fig. 7. After the preparation of sections, a Phenocycler run was executed. Mouse femur cryosections of 10 μm thickness were acquired from O. C. T.-embedded material on slides treated with silane or coated with Cell-Tak™. We compared both types of slides since we wanted to test which condition gives us the best possible result with respect to tissue adhesion as well as imaging. Sections were prepared following the protocol provided by the manufacturer, Akoya Biosciences, which was also described in detail by Goltsev et al. [22]. An antibody master mix described below was utilized. A pre- and post-stain fixation was utilized. Slides were mounted with a proprietary flow cell from Akoya Biosciences and imaged using the Phenocycler 2.0™ system. The workflow for the Phenocycler staining and post-staining steps is shown in Supplemental Fig. 8.

## Reporter plate setup

The corresponding oligo-barcodes with reporter probes were added by the automated system of the Phenocycler 2.0^TM^ during each cycle. The DNA-DNA bonds of reporter oligos were denatured at the end of each cycle to facilitate the subsequent addition of a new set of oligo-fluorophore reporters. The reporter probes were added to the wells of a black round-bottom low-binding 96-well plate. The reporter stock solution was added per well, minus the volume of the reporter probes. The workflow for setting up the reporter plate is shown below:

The reporter stock solution was prepared for the total number of cycles for each Phenocycler run by utilizing the volumes given in Table 5.

**Table 5:** Volumes for the reagents to make the reporter stock solution.

	1 cycle
Nuclease-free water (µl)	244
10× Phenocycler Buffer (µl)	30
Assay reagent (µl)	25
Nuclear Stain (µl)	1
Total Volume (µl)	300

Each reporter plate was set up by adding 250 µl of reporter stock solution minus the volume of the oligo-barcode probes (5 µl per probe) added per well. For example: If well 1 has no probes, then the volume of reporter stock solution to be added to well 1 was 250 µl. If well 2 had 2 oligo-barcode probes, then the volume of reporter stock solution was 250- (5 × 2) µl = 240 µl. Since there are 3 channels available per cycle, the maximum number of probes that can be added per well is 3, hence, the total volume in the well would be 235 µl reporter stock solution + 15 µl total volume of probes (5 µl each). All wells containing reporter solution were sealed with adhesive aluminum strips to prevent evaporation before there are automatically dispensed to the stage by the Phenocycler 2.0™ fluidics handling system.

### Designing the Phenocycler run and experimental setup on the instrument

Runs were configured as an experimental template in Akoya Phenocycler Experiment Designer™ (Akoya PED™) software, and then this configuration was loaded at the start of the run. The template for the cycles with the pre-run and interleaved blanks is shown in Supplemental Fig. 3. Akoya uses a blank at the beginning and a blank at the end to mathematically approximate the amount of autofluorescence in each round of imaging. We found that autofluorescence varied enough that this procedure gave inadequate results, and so we collected autofluorescence images every other round. To allow for the background subtraction for each marker, we set up the blanks corresponding to their respective markers set at exposure times that were the same as the markers. We also found that autofluorescence increased logarithmically as cycle number increased, an effect that could be minimized by imaging the unlabeled tissues several times before the addition of markers to reduce variation in autofluorescence during the portion of the run that introduces oligo-fluorophores. To accommodate this, we set up the pre-run blanks (pre-treat cycles) at exposure 150 ms for the autofluorescence to plateau the change in autofluorescence signal before the markers were added or blanks collected. Because of software constraints, we still needed to set a blank as the first and last cycles. These blanks are set up in wells H1 and 2 on a 96-well plate.

### Multiplex data processing and background subtraction

Instead of utilizing the ‘.qptiff’ image files generated automatically by Akoya Biosciences®, a custom workflow was developed. Raw data was generated in a ‘temp’ folder during data acquisition. The ‘.raw.qptiff’ files were stitched using the Akoya Biosceinces® algorithm but do not have any background correction applied. The ‘raw.qptiff’ is then post-processed in ImageJ/ FIJI (referred to as FIJI henceforth) [29]. as shown in Supplemental Fig. 2. Preceding each cycle, an empty cycle was run to generate a blank for each marker in the antibody panel, details of the setup are shown in Supplemental Fig. 3. ‘.raw.qptiff’ files from each Phenocycler 2.0™ run were cropped on import at the highest resolution setting using FIJI Within FIJI, the Image Calculator Subtract function was used to remove the blank image associated with each marker. The resulting subtracted images for each individual marker were then imported into HALO™ (Indica Labs) and fused with all other markers generating a multiplex ‘.afi’ file. The fused images were used for segmentation for cell type identification. The High-Plex FL module was configured to identify critical phenotypes by establishing thresholds for positivity for each marker. Each phenotype is described in Table 1. Segmentation parameters and cut-off thresholds were iteratively analyzed in the real-time tuning window to lay a mask over the cells identified for the phenotype. These segmentation parameters were then applied equally to each tissue, and cell object data was exported to a table for quantification. MKs were segmented differently than the other cell types due to their size and polyploid nucleus.

### Comparison of data from Phenocycler 2.0™ imaging and flow cytometry

**Fig. 2:**
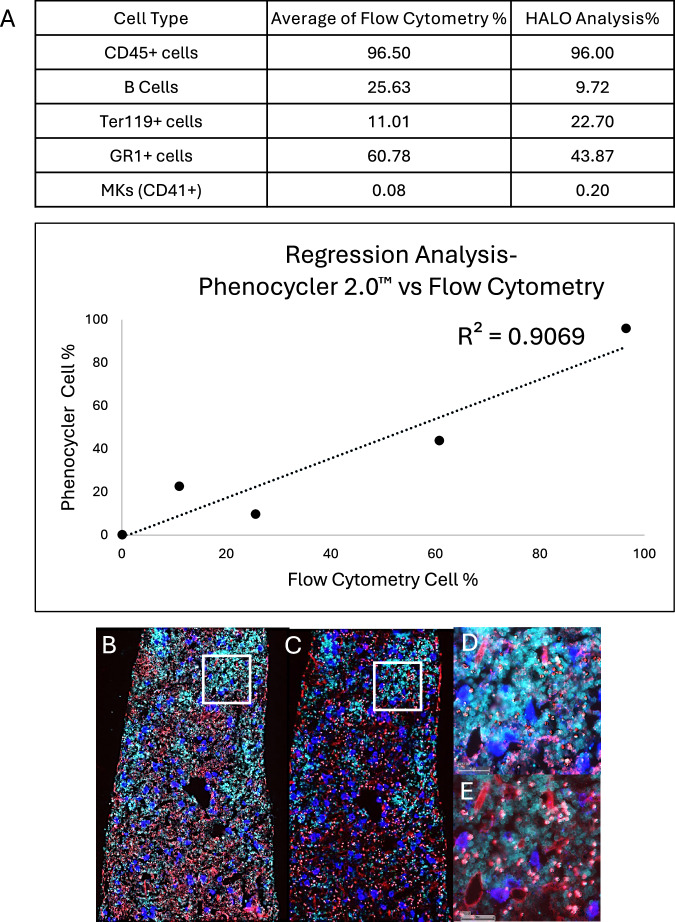
Comparison of imaging results obtained by using built-in image processing vs our imaging pipeline. **A** Regression analysis showing the comparison between cell percentages obtained by HALO™ analysis using Phenocycler 2.0^TM^ images vs cell percentages from flow cytometry of C57BL/6J femurs for CD45, B220, Ter119, GR1, and CD41 markers. Difference between the final image output using **B** built-in image processing and Phenochart™ (QPTiff) vs **C** image obtained by our protocol using ‘.raw.qptiff’ files and manual background subtraction using FIJI of the same tissue. Markers shown are CD31 SCA1 Endomucin (all red), GR1 (cyan) and CD41 (blue). **D** Higher-magnification image of the area shown in white square from built-in image processing. Scale bar 50 µm. **E** Higher-magnification image of the area shown in white square from image obtained by our protocol. Scale bar 50 µm. Signal from GR1 and CD41 markers was over-saturated in QPTiff image (**B**) and the cell types could not be segmented for HALO™ image analysis.

BM region was first annotated using the annotation function in HALO™, and then this region was used to obtain the total number of nucleated cells. After applying segmentation parameters as described in the previous section, the number of cells for the main cell types of the BM were obtained. The cell percentages were calculated as:

(number of cells identified by segmentation parameters of that cell type in BM/ total number of cells in BM) × 100. The cell percentages derived from the HALO™ analysis were then compared to the flow cytometry cell percentages of that cell type. A regression analysis was performed to see the level of concordance between the cell percentages of the two modalities compared to each other (Fig. 2A).

## Results

### Regression analysis for comparison of cell percentages from Phenocycler 2.0™ and flow cytometry

Flow cytometry is a well-established technique and is used extensively to study the cell composition of the BM. To check the accuracy of our Phenocycler 2.0™ imaging and HALO™ image analysis results, the cell percentages obtained from Phenocycler 2.0™ imaging and flow cytometry were compared. The process of obtaining cell percentages is described above. Markers that were selected for this comparison were CD45, Ter119, B220, Gr1, and CD41 since they cover the major cell types of the BM as shown in Table 1. Regression analysis showed that the correlation coefficient was 0.90 (Fig. 2A). We observed the highest degree of concordance between the frequency of CD45, followed by Ter119 and GR1 markers, utilizing flow cytometry and Phenocycler 2.0™. However, the concordance was lower for B220 and CD41 markers compared to CD45, Ter119, and GR1. While the cell percentages compared relatively well in some cell types, certain cell types, such as CD41+ MKs, were challenging to segment due to the limitation of the HALO™ analysis software to handle polyploid nucleated cells. The differences noted between the two modalities of measuring the cell percentages could be attributed to the techniques being inherently different (imaging a tissue section vs fluorophore detection on cells that are mostly in a single-cell suspension). Newer versions of the HALO™ software might have better flexibility to detect unusually large or small cells that have different nuclear presentations. Even though some markers had lower concordance than others, the agreement between the two modalities of analysis was higher than the cell percentages obtained using HALO™ from manufacturer built-in image processing. Using the manufacturer built-in image processing, some markers such as CD41, B220, and GR1 appeared over-saturated to the extent that the cells could not be segmented accurately in HALO™ (Fig. 2B–E).

We were unable to directly compare our custom post-processing pipeline to the standard Akoya Biosciences® image processing due to several factors. Pixel intensity saturation was frequently observed confounding thresholding attempts. Segmentation attempts were further disrupted by the high level of background fluorescence observed.

### Cell and structural markers identified

**Fig. 3:**
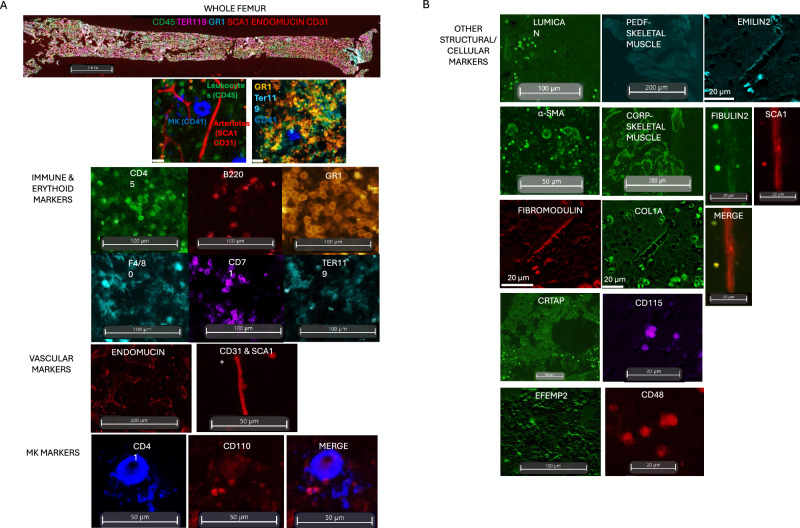
Images of different cell and structural markers in murine femur on Silane-treated slide from Phenocycler 2.0^TM^ imaging. **A** Whole femur showing markers CD45, TER119, GR1, SCA1, Endomucin, and CD31. Scale bar: 1 mm. Inserts below show higher-magnification multiplex images of different cell types and arterioles. Scale bar: 20 µm. Individual markers for cell surface antigens and vascular structures at higher magnification are shown below the multiplex images. **B** Other structural and cellular markers shown at higher magnification.

Figure 3 shows whole femur multiplex images along with the different individual cell and structural markers from a C57BL/6J mouse femur. Similar results were seen in 4 runs for the C57BL/6J mouse femur. Figure 3A shows the whole femur, which was stained for CD45, Ter119, Gr1, SCA1, CD31, and Endomucin to demonstrate the overall structure of the murine BM on a Silane-treated slide. Inserts below show multiplex images at higher magnification. The higher magnification multiplex image on the left shows arterioles (CD31 and SCA1 positive, red) as well as MK (CD41, blue), and leukocytes (CD45, a pan-leukocyte marker, green). The higher-magnification multiplex image on the right shows the lineage markers, GR1 (orange) and Ter119 (cyan), as well as MK (CD41, blue). We also observed that the lineage markers (GR1 and Ter119) did not colocalize in tissues, confirming that the signal from these markers is specific and identifies the correct cell type. Individual cell and structural markers from the whole femur are shown at a higher magnification in the inserts. CD45 (pan-leukocyte marker, shown in green), along with immune cell markers such as B220 (red), GR1 (orange), and F4/80 (cyan), can be seen in the inserts below the whole femur. Erythroid cell markers, including Ter119 (cyan) and CD71 (magenta) are also shown in the panel of inserts below the whole femur. As seen in these images, the labeling of these cell markers is bright and specific to the cell type. MKs are large cells and are identified by CD41 (blue) positive staining [30]. CD110 (red), thrombopoietin receptor, is critical for MK proliferation and is present on MKs as well as some other cells in BM such as stem and progenitor cells and platelets [31, 32]. CD110 is shown colocalizing on MKs with CD41 in the merged image (red + blue).

Vascular markers such as Endomucin (red), which labels sinusoids [33, 34], and CD31 (red), along with SCA1 (red), which label different cells of arterioles such as endothelial cells and endothelial progenitors [35], can be seen in Fig. 2A, highlighting the vascular structures. Fibulin2 (green), which is another structural marker found in the extracellular matrix (ECM) as well as the basement membrane of different cells in the BM [36], can be seen colocalize with SCA1 (red) in arterioles, as seen in Fig. 3B insert (merged image (red + green)).

Figure 3B also demonstrates the presence of additional BM-associated structural markers. Lumican (green) is secreted by osteoblasts in the BM and is found in the ECM of the BM, bone, and skeletal muscles abundantly [37, 38]. Emilin2 (cyan) is secreted by mesenchymal stem cells (MSCs) in the ECM of BM [39, 40]. EFEMP2 (green) is a secreted protein present in the basement membrane of cells of connective tissues such as the BM and skeletal muscle [41, 42]. Fibromodulin (red) is expressed by BM stromal cells and osteoblasts and is found in the ECM of BM [43, 44]. markers [40, 42, 44]. We observed lumican (green), Emilin2 (cyan), EFEMP2 (green), and Fibromodulin (red) positive signals throughout the ECM of the BM. PEDF (cyan), a factor secreted by MSCs in the BM [45–47], was seen abundantly in the skeletal muscle attached to the bone and the BM. α-SMA (green) is a cytoskeletal protein that is present in the cytoplasm of cells [48] and is readily detected in almost all cells of the BM. Col1a (green) is the most common type of collagen [49] in the body and is detected in abundance in the BM, as well as adjacent tissues such as skeletal muscle. CRTAP (green) is a protein that is associated with post-translational collagen modifications in articular cartilage and bone and is usually seen near the growth plates of long bones [50]. CRTAP was observed abundantly in the epiphyseal region of the femur. CGRP (green) is found in the BM, bone, and skeletal muscle [51] and was seen abundantly in the skeletal muscle and bone. Figure 3B also shows other cell markers such as CD115 (magenta) and CD48 (red). CD115 (also known as colony stimulating factor 1 receptor) is a cell surface marker found on myeloid lineage cells such as monocytes, macrophages, and osteoclasts (which differentiate from monocyte lineage cells) [52] was observed on cells that were closer to endosteum (region of BM close to the bone). Even though the cells staining positive for CD115 were not abundant, the marker produced a bright signal on these cells. CD48 is a cell surface marker that is expressed by different progenitors such as myeloid-erythroid and B lineage progenitors, however, CD48 is not expressed by multipotent progenitors or primitive cells such as HSCs. Cells that were positive for CD48 (red) could be seen throughout the BM.

### Comparison of tissue adhesives: - Cell-Tak™ coated slides vs. Silane-treated slides

Tissue adhesion is of critical importance to the success of imaging using Phenocycler 2.0™ since the tissue is subjected to serial washes between imaging steps. Poor anchoring of the tissue to the slide during processing can severely compromise the image including resulting in tissue lifting off the slide, which can cause issues with focus. We found that both the tissue adhesives, Cell-Tak™ and Silane, provided sufficient tissue adhesion for BM imaging, although some differences were noted.

Cell-Tak™ is a tissue and cell adhesive that contains an adhesive protein from the common blue mussel (*Mytilus edulis L*.). Cell-Tak™ is used to increase adhesion for whole tissue sections on slides or cells in in vitro conditions [53–55]. Silane-treated slides are routinely used for the adhesion of cells [56–58].

**Fig. 4:**
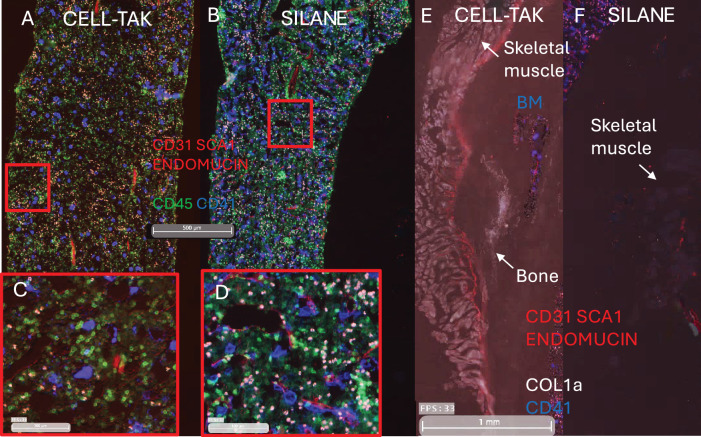
Comparison between Cell-Tak™ and Silane as tissue adhesives. **A** Murine femur from C57BL/6J on Cell-Tak™ coated slide at lower magnification and **B** Murine femur from C57BL/6J on Silane-treated slide showing lymphocytes (CD45 positive), vascular structures (CD31, SCA1, and Endomucin positive) and MKs (CD41 positive) at lower magnification. Scale bar: 500 µm. **C** Murine femur from C57BL/6J on Cell-Tak coated slide from region shown in red on Cell-Tak™ coated slide at higher magnification and **D** Murine femur from C57BL/6J on Silane-treated slide from region shown in red on Silane-treated slide at higher magnification. Scale bar: 100 µm. Both tissue adhesives performed adequately during CODEX runs. **E** Murine femur from C57BL/6J on Cell-Tak™ coated slide and **F** Murine femur from C57BL/6J on Silane-treated slide showing vascular markers (CD31, SCA1, Endomucin), CD41, and Col1a. Scale bar: 1 mm. Cell-Tak™ coated slide was able to retain tissues adjacent to bone marrow, such as bone matrix and muscles, better (**E**) than the Silane-treated slide (**F**) as the Silane-treated slide had few areas of the adjacent tissues and showed some Col1a staining and skeletal muscle shown by the arrow.

As shown in Fig. 4, the lower and higher-magnification images highlight the differences between the images obtained using two tissue adhesives in a Phenocycler 2.0™ run. Cell and vascular markers such as CD31, SCA1, Endomucin (all in red), CD41 (blue), and CD45 (green) were selected to show the general architecture of the BM (Fig. 4A–D). The adjacent tissues, such as skeletal muscle and bone, were shown by Col1a (white) since Col1a is abundant in these tissues (Fig. 4E, F).

The general architecture of the BM at lower magnification on a Cell-Tak™ coated slide (Fig. 4A) and a Silane-coated slide (Fig. 4B). Figure 4C shows the boxed area in Fig. 4A at higher magnification. Similarly, Fig. 4D shows the boxed area in Fig. 4B at higher magnification. As seen in Fig. 4A–D; both the tissue adhesives show strong BM adherence to the slide as measured by the lack of tissue lifting and subsequent focus issues. The BM structurally in these images looks intact and remains on the slide surface. Figure 4C, D also shows the vascular structures shown by markers CD31, SCA1, and Endomucin in red along with MKs (CD41, blue) and BM cells positive for CD45 (green). However, as seen in Fig. 4E, F, Cell-Tak™ was better at preserving the adjacent tissue such as bone and skeletal muscle attached to the bone (seen labeled by Col1a in white). Figure 4F shows a Silane-treated slide with some skeletal muscle shown by the arrow and no bone. These observations are important to note since the choice of tissue adhesive would impact the Phenocycler imaging for tissues of interest in a study. For example, Silane or Cell-Tak™ would both be suitable to image the BM cells and structures; however, only Cell-Tak™ would be suitable to study the BM adjacent tissues such as bone and skeletal muscles since Silane-treated slides did not adhere these tissues well.

## Phenocycler 2.0™ imaging of murine wild type and MK ablation model femurs

**Fig. 5:**
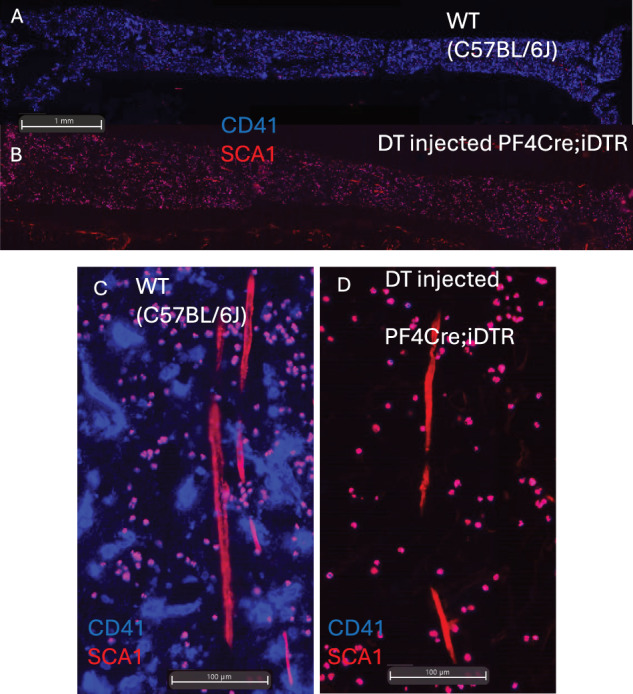
Differences between bone marrow of a wild-type mouse and inducible MK ablation Cre mouse model shown by Phenocycler 2.0^TM^ multiplex imaging. Phenocycler 2.0^TM^ imaging of **A** C57BL/6J and **B** DT-injected PF4Cre; iDTR femurs at lower magnifications. **C** C57BL/6J and **D** DT-injected PF4Cre; iDTR femurs at higher magnifications. PF4Cre; iDTR is an inducible Cre model to ablate MKs. SCA1 was selected as a marker to show vasculature and the differences between the mouse models and highlight the drastic reduction in the MKs. **C** CD41, a marker for MKs and platelets is seen in blue in the femur of a C57BL/6J mouse along with SCA1, showing arterioles. **D** CD41 marker is not seen in the femur of a DT-injected PF4Cre; iDTR mouse, suggesting ablation of MKs.

Figure 5 shows the differences between the MKs in male C57BL/6J (wild type, WT) femur and Diphtheria toxin (DT) injected PF4 (platelet factor 4) Cre; iDTR (inducible diphtheria toxin receptor) mouse femur. PF4Cre; iDTR mouse is an inducible mouse model to ablate MKs and platelets when DT is injected [59]. This mouse is generated by crossing PF4Cre mice with iDTR homozygous mice. When injected with DT, the DT binds specifically to the induced receptors on MKs in the PF4Cre; iDTR mice and ablate the MK populations [59, 60]. This mouse model is used to study the effects of MKs and platelets in BM microenvironments or to study the effects of different treatments in MK ablated conditions [59, 61]. The PF4Cre; iDTR mice for the Phenocycler 2.0™ imaging experiment were injected with a higher dose of DT (100 ng/ ml; 2× weekly) to show the drastic depletion of the MKs and, in turn, CD41, which is an MK marker. Figure 5 highlights the differences between the WT (Fig. 5A) and DT-injected MK ablated femur (Fig. 5B). CD41 (blue) and SCA1 (red) were the only 2 markers selected to show the drastic differences in the loss of MKs upon DT treatment of this mouse model. Even though rare (0.01–0.02% of BM), due to their size, MKs appear as large cells in the WT femur at lower magnification. CD41 is also a marker for platelets. Platelets can be found closer to the arterioles when the MKs release them. Together, MKs and platelets are seen throughout the WT femur and appear to dominate the tissue. Higher-magnification image of the WT femur (Fig. 5C) shows the CD41 stained MKs and adjacent SCA1 stained arterioles. Figure 5D shows no CD41 staining and only SCA1 positive staining for arterioles, suggesting MK ablation. It is important to note that both the femur sections (WT and DT-injected PF4Cre; iDTR femurs) were imaged on the same slide and were stained and imaged on Phenocycler 2.0™ at the same time. This eliminates the possibility of experimental variation as the cause of the depletion.

## Pheocycler 2.0™ imaging of primitive and more committed progenitor cells in a murine wild-type femur

**Fig. 6:**
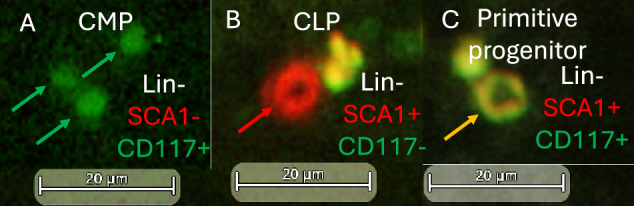
Phenocycler 2.0^TM^ imaging of C57BL/6J showing CMP, CLP, and HSC cell populations. **A** CMP cells were identified as Lineage- SCA1- CD117+. **B** CLP cells were identified as Lineage- CD117- SCA1+. **C** Primitive progenitors were identified as LSK (Lineage- SCA1 + CD117+). Scale bar: 20 µm.

We next attempted to identify more primitive stem and progenitor cells of the BM. Lineage negative (Lin-) is defined as negative for B220, GR1, and Ter119. As seen in Fig. 6A, we readily identified Lin-, SCA1+, and CD117+ also known as LSK cells of the BM. These are a population of BM cells that contain the most primitive fraction of long-term initiating stem cells. Figure 6B shows the presence of Lin-, SCA1-, and CD117+ cells, and Fig. 6C demonstrates the presence of Lin-, SCA1 + , CD117- fraction of more primitive cells. A CMP cell is a multipotent cell that can differentiate into MK-erythrocyte progenitor (MEP) or granulocyte-monocyte progenitor (GMP) [10, 62, 63]. As shown in Fig. 6B, CMP cells were identified as Lin-, SCA1-, CD117+ cells. A CLP cell can differentiate to form lymphocytes such as T, B, and natural killer cells [9, 63, 64]. As shown in Fig. 6C, CLP cells were identified as Lin-, CD117-, SCA1+. LSK cells are rare multipotent cells or primitive progenitors that can differentiate into all types of blood cells, such as CMP and CLP as well as consist of self-renewing stem cells (primitive progenitors) [65].

## Limitations

Phenocycler 2.0™ is a useful tool for achieving multiplexed imaging of tissues that need modalities that can surpass the limited number of markers available traditionally. However, it is also complex due to the inclusion of multiple markers in a panel that have differential expressions in the tissue of interest. For example, some antibodies (either conjugated by the user or purchased commercially) might generate a very bright signal that is well above the noise from the tissue autofluorescence. However, some antibodies might be dim due to either the nature of the epitopes or the rarity of the markers themselves. This is usually not the limitation of traditional techniques, such as IF, which can image ~3+ markers in one imaging run. In IF, the conditions needed to obtain the optimal signal from an individual marker can be applied to the tissue and can be detected without any need for special software.

The addition of “pre-treat” and interleaved blanks for individual markers added significantly to the run time. With the expansion of the panel to include more markers, the number of interleaved blanks will also increase, in turn leading to longer run time on the Phenocycler 2.0™ instrument. The manual background subtraction using ‘.raw.qptiff’ files and FIJI is also time-consuming compared to the built-in image processing from the manufacturer using Phenochart 2.0™ to visualize the image. While longer run time on the instrument is a significant limitation, the images that were obtained were more accurate than the built-in software generated QPTiffs, especially in visual representations of some markers such as CD117, CD41, α-SMA, CD48, Endomucin, and many of the ECM markers.

Another limitation is the presence of autofluorescent particles or bodies that are not cells (these are enucleated) and are ubiquitous in the BM. We were not able to get rid of these autofluorescent particles by either photobleaching or by treating the sections with bleaching agents such as hydrogen peroxide at different concentrations (data not shown). Even though these particles do not affect the cell segmentation and identification as they can be eliminated from the HALO™ analysis, these can be very distracting, especially to the human eye when identifying different cells close to these autofluorescent particles. A recent study done by Bandyopadhyay et al*.* also observed similar autofluorescent particles in the BM of humans [21]. The authors labeled these particles with human anti-mast cell tryptase for identification since these particles stained positive for almost all the markers. Further studies need to be done to identify the true nature of these autofluorescent particles as well as to either dampen their signal or eliminate them from the images completely.

We have used cryosectioned femurs as our tissues to stain for the Phenocycler 2.0™ imaging of the murine BM. However, for this tool to be widely used for studying and interrogating different disease states and physiological conditions, it needs to be adapted for formalin or PFA-fixed murine tissues with higher autofluorescence. High autofluorescence in formalin or PFA-fixed tissue develops due to the crosslinks that form in proteins post-fixation in aldehyde-based fixatives. Our observation of some of the markers used to identify the progenitors and main cell types of murine BM shows that some of these markers produce dim signals (such as CD117). These markers might be challenging to adapt to the fixed and paraffin-embedded tissues without major modifications to the protocol we recommend. We have used young male mice (12–15 weeks old) for our protocol. It would be particularly interesting to see if this protocol can also be used to image the cryosections from older mice since older tissues often have higher autofluorescence due to the accumulation of lipofuscin [66–68].

Despite the above-mentioned limitations, Phenocycler 2.0™ is still a desirable multiplex imaging tool that can not only image rare cell types and give users a means for conducting in-depth cellular analyses, but it is also the only multiplex imaging platform that can recover the tissue at the end. The recovered tissue can be re-run without the need to be stained again, or other histochemical analyses can be performed, such as H&E staining. Our protocol covers a broad panel of antibodies that cover several markers for immune cells, hematopoiesis, vasculature, and ECM. However, this panel can be expanded by other researchers using the information in our study. One of the biggest advantages of using Phenocycler 2.0™ is that markers that have commercially available antibodies can be custom-conjugated and used in conjunction with either the existing pre-conjugated commercial antibodies or other user-developed panels (such as this protocol).

## Conclusion

Phenocycler 2.0™ can be a particularly useful tool to study unperturbed BM with the cell neighborhoods and structures intact. It can be a powerful analytical tool when combined with an informatics tool such as HALO™ image analysis software to study cellular interactions and how they change in the knock-out or knock-in murine models, which can help develop therapies for different diseases. However, the accuracy of the visual representation of the markers needs to be scrutinized and cross-checked with established techniques such as IF. We were able to develop a protocol from tissue processing steps to image processing and analysis, which was able to provide accurate visualizations of the cell and structural markers.

## Supplementary information

Supplemental Material

Supplemental Figures

## Data Availability

The datasets generated during and/or analyzed during the current study are available from the corresponding author on reasonable request.
