# Supplementary material for: Multiplex imaging of murine bone marrow using Phenocycler 2.0™
Source: Leukemia. 2025 Apr 11;39(6):1476–89. doi: 10.1038/s41375-025-02596-5 (PMC12133563; doi:10.1038/s41375-025-02596-5)
Supplement: Supplementary file 1 — Supplemental Material [file 41375_2025_2596_MOESM1_ESM.docx]

**Troubleshooting**

Steps to address issues arising during tissue processing or setting-up the run and the potential solutions are listed in **Supplemental** **Table 1.**

**Supplemental Table 1. Troubleshooting table.**

| **Step** | **Problem** | **Possible reason** | **Solution** |
| --- | --- | --- | --- |
| Tissue cryosectioning. | Tissue has tears. | Blade not sharp. | Change blade. |
|  |  | O. C. T. has not penetrated the tissue. | Embed the tissue in O. C. T. and place the cryomolds at 4°C, then at -20°C (till O. C. T. solidifies), and finally store them at -80°C. |
|  | Tissue not adhering to the slide after Cell-Tak™ coating. | Cell-Tak™ has expired. | Cell-Tak™ has a limited shelf-life. Use new vial of Cell-Tak™. |
|  |  | Slide were coated and stored longer than recommended storage duration (14 days) and/ or at wrong temperature (higher or lower than 4°C, for long term storage). | Coat slides with Cell-Tak™ closer to Phenocycler run. Store coated slides at recommended temperature and for recommended duration. |
|  |  | Stock solution of sodium carbonate or bicarbonate is old and has changed pH. | Check solutions used to dilute and neutralize the Cell-Tak™ stock solution. Prepare fresh solutions to dilute and neutralize Cell-Tak™ stock. (See manufacturer instructions for preparing stock solutions used for diluting Cell-Tak™). |
| Staining steps. | Tissue lift-off during staining steps. | Problems with cryosectioning. | Check expiration for folds and only use tissue that is free of folds and creases. |
|  |  | Problems with tissue adhesion on the slide. | For Cell-Tak™ coated slides, check Cell-Tak™ expiration date. See solutions listed above in Step: Tissue cryosectioning. |
|  |  |  | For Silane treated slide, check for folds, and only use tissue that is free of folds and creases. |
|  |  | Handling slides and pipette tips scratching the tissue during pipetting steps. | Be gentle when pipetting solutions on the tissue. Avoid direct contact of the pipette tip with the tissue. |
| Image processing. | Marker has low visibility. | Exposure time is low. | Increase exposure time. |
|  |  | Marker is rare and was assigned to 750 nm channel. | Assign the marker to a brighter channel (550 nm). |
|  |  | Clone used is not compatible for the protocol. | Check the IF validation results. If the clone performed poorly in IF validation step, change the clone used and re-validate. |

**Step by step protocol and timing**

Times reported are for the staining, image processing, and analysis steps for Phenocycler 2.0™ run and HALO™ analysis. The time taken for tissue processing and sectioning are dependent on the number of tissues.

Step: Staining

Day 1: 50 minutes to 1 hour.

Day 2: 45 minutes to 1 hour.

Step: Imaging on Phenocycler 2.0™ system

Establishing the experiment design parameters: 10- 15 minutes.

Placing slide with tissues on the imaging platform and testing instrument for leaks: 30 minutes

Actual run: 45 minutes x number of cycles (for the panel in our protocol, ~24 hours.)

Instrument post-run clean-up prompted by the software: 10 minutes.

Step: Image processing and background subtraction using FIJI

Image processing using our protocol: 4-5 hours for image extraction and subtraction with corresponding blanks (for entire panel in our protocol).

This time may vary depending on the number of markers used in a run. Additionally, data management and transfer will add variable time to this process depending on the total size and transfer method utilized.

Step: HALO 4.0™ image analysis

Image analysis on HALO™: 5-6 hours

This time is based on the markers we selected for HALO™ analysis (CD41, CD45, Ter119, B220, and GR1). The time may also vary depending on the quality of staining, the ease of segmentation of the tissue.

**Supplemental Figures**

**S1.** Presence of autofluorescence due to RBCs and comparison of perfused and non-perfused femurs. (A) Autofluorescent RBCs in 550 nm channel, (B) Autofluorescent RBCs in 647 nm channel, and (C) Autofluorescent RBCs in 750 nm channel. Scale bar: 20 µm. Even though the RBCs fluoresce to a lesser extent in 750 nm channel, they were visible but dim. (D) Perfused femur showing empty spaces due to probable tissue loss. Perfusion led to the loss of vascular structures, even though, not an entire loss of the signal from CD31+ and SCA1+ cells. (E) Non-perfused femur had well-preserved vasculature shown here as SCA1+ and CD31+ capillary. Non-perfused femur had better tissue integrity compared to the perfused femur. Scale bar: 200 µm.

**S2.** Comparison of workflows Akoya Biosciences® image processing pipeline and customized image processing pipeline with interleaved blanks and image processing using .raw.qptiff files generated after the completion of Phenocycler 2.0™ run. (A) Output image from Akoya Phenocycler 2.0™ image processing pipeline (QPTiff) viewed using Phenochart ™ showing saturated signal from markers CD31, SCA1, and Endomucin (all red), CD41 (blue) and α-SMA (green). (B) Output image using our image processing described in our protocol of the same tissue showing specific and clear labeling of CD31, SCA1, and Endomucin (all red), CD41 (blue) and α-SMA (green).

**S3.** Experimental set-up for Phenocycler 2.0™ run showing pre-run (pre-treat) blanks along with interleaved blanks for the respective markers. Pre-treat blanks (first 7 cycles without fluorescent reporter oligo-barcodes) were added for the autofluorescence to plateau before the actual cycles with antibodies start. The interleaved individual blanks corresponding to the cell or structural marker were added to get possible background subtraction using the ‘.raw.qptiff’ images. The blanks according to Akoya Biosciences® must still be set-up for the system to run.

**S4.** Representative images of gel electrophoresis of conjugated antibodies verifying the conjugation of the oligo-barcode onto the antibody. The unconjugated antibody shows a light chain and a heavy chain band (red circles). After conjugation, there is a shift in the band size due to the addition of oligo-barcode via a maleimide bond and the band splits into multiple bands (indicated by blue squares).

**S5.** Autofluorescence in murine bone marrow samples. Different fixatives and embedding techniques have different effects on the autofluorescence of murine bone marrow samples. Cryosectioned OCT-embedded bone marrow samples have reduced autofluorescence compared to paraffin-embedded samples. Methanol: Acetone (1:1) showed the least autofluorescence in the cryosectioned samples. Scale bar: 1000 µm.

**S6.** Representative images of IF validations of in-house conjugated antibodies. Green shows the conjugated antibody and blue is DAPI for nuclear stain. Scale bar: 100 µm.

**S7.** Workflow for executing steps for different stages in tissue and custom-conjugated antibodies for Phenocycler 2.0™ run.

**S8.** Workflow for staining the cryosectioned tissue for Phenocycler 2.0™ run.
