# Supplementary material for: Multiplex imaging of murine bone marrow using Phenocycler 2.0™
Source: Leukemia. 2025 Apr 11;39(6):1476–89. doi: 10.1038/s41375-025-02596-5 (PMC12133563; doi:10.1038/s41375-025-02596-5)
Supplement: Supplementary file 2 — Supplemental Figures [file 41375_2025_2596_MOESM2_ESM.pptx]

## Slide 1
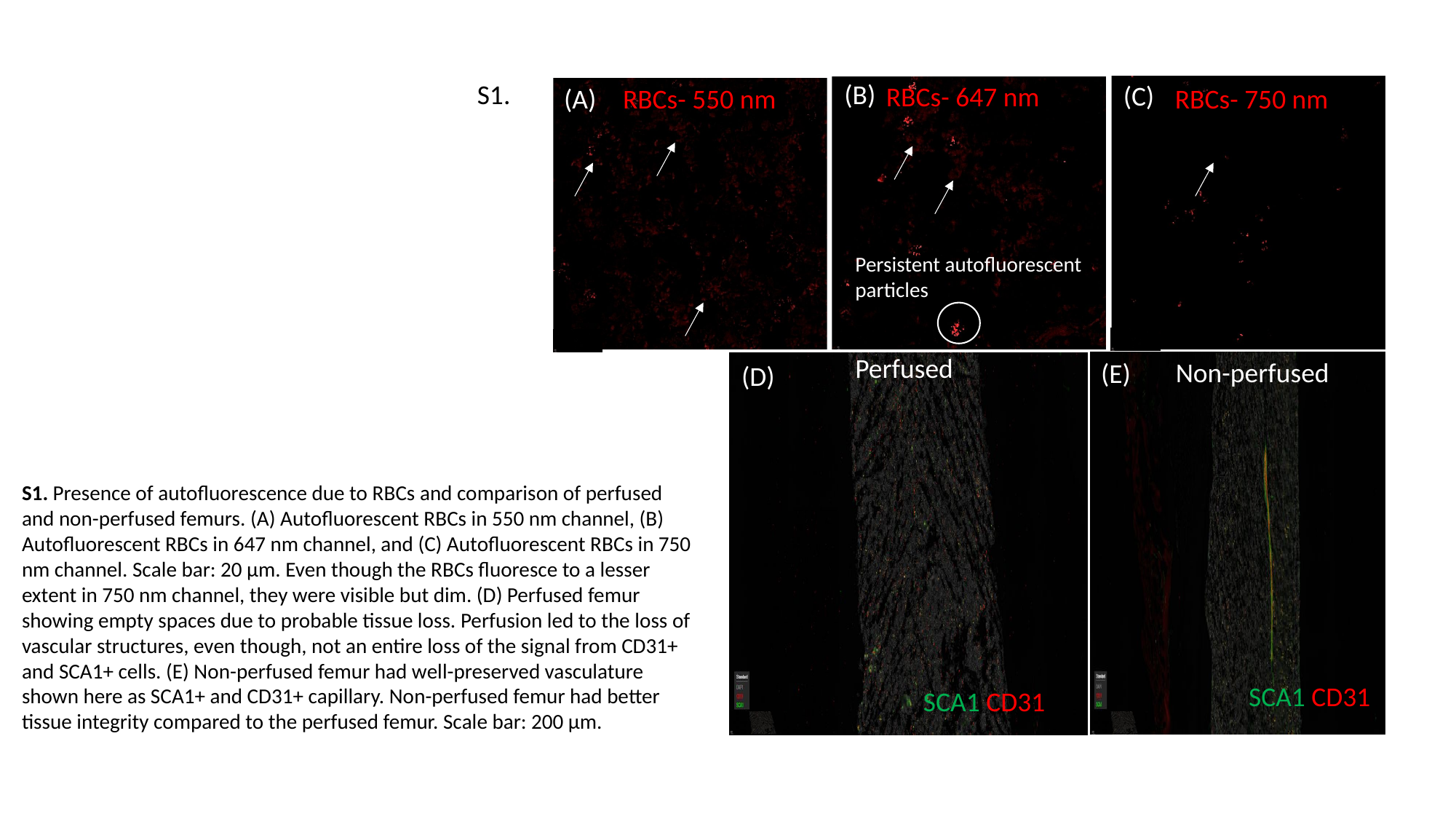

S1.
(B)
(C)
RBCs- 647 nm
Perfused
Non-perfused
SCA1 CD31
SCA1 CD31
RBCs- 550 nm
RBCs- 750 nm
(A)
(E)
(D)
Persistent autofluorescent particles
S1. Presence of autofluorescence due to RBCs and comparison of perfused and non-perfused femurs. (A) Autofluorescent RBCs in 550 nm channel, (B) Autofluorescent RBCs in 647 nm channel, and (C) Autofluorescent RBCs in 750 nm channel. Scale bar: 20 µm. Even though the RBCs fluoresce to a lesser extent in 750 nm channel, they were visible but dim. (D) Perfused femur showing empty spaces due to probable tissue loss. Perfusion led to the loss of vascular structures, even though, not an entire loss of the signal from CD31+ and SCA1+ cells. (E) Non-perfused femur had well-preserved vasculature shown here as SCA1+ and CD31+ capillary. Non-perfused femur had better tissue integrity compared to the perfused femur. Scale bar: 200 µm.

## Slide 2
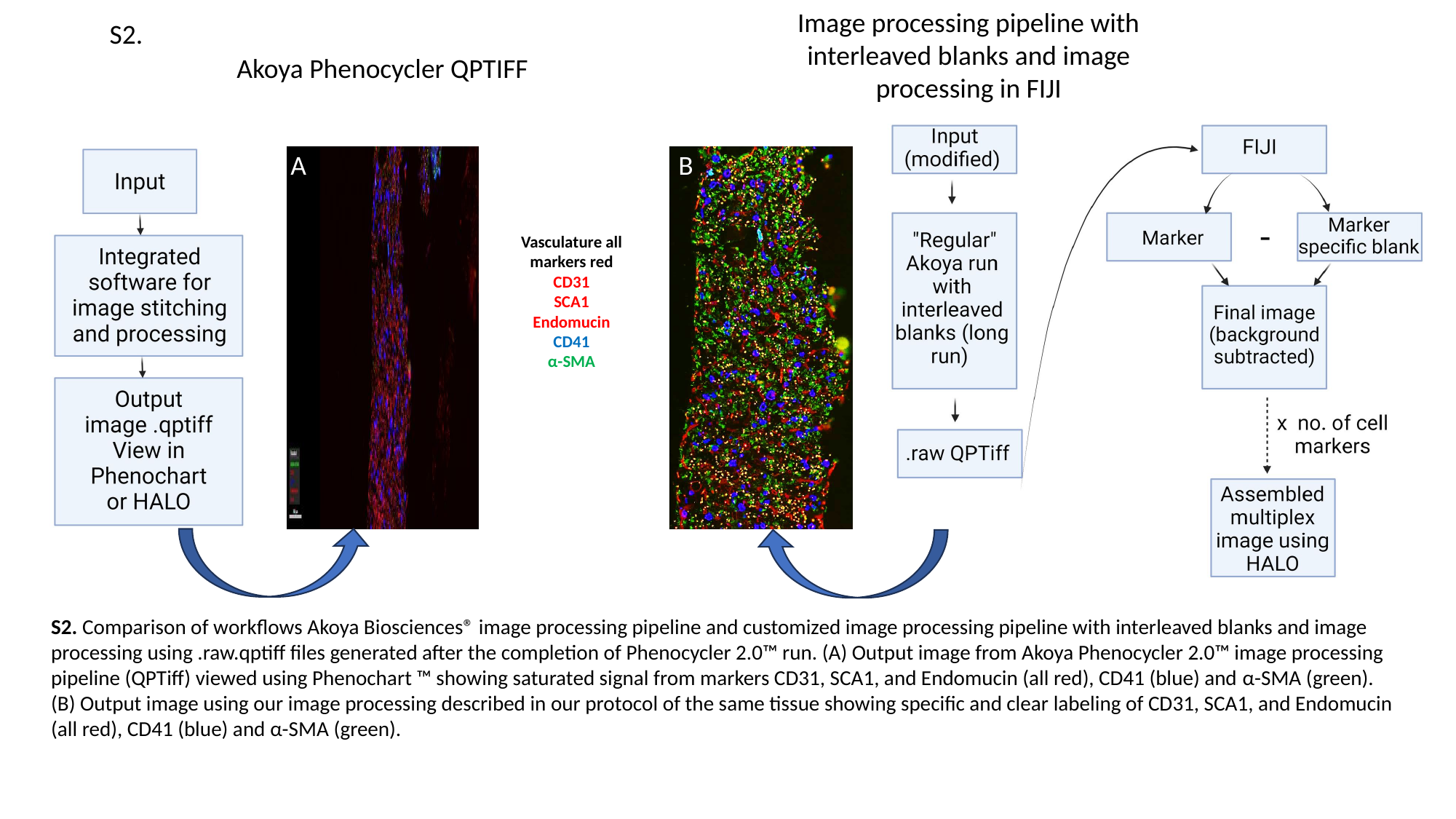

Image processing pipeline with interleaved blanks and image processing in FIJI
Akoya Phenocycler QPTIFF
A
B
Vasculature all markers red
CD31
SCA1
Endomucin
CD41
α-SMA
S2.
S2. Comparison of workflows Akoya Biosciences® image processing pipeline and customized image processing pipeline with interleaved blanks and image processing using .raw.qptiff files generated after the completion of Phenocycler 2.0™ run. (A) Output image from Akoya Phenocycler 2.0™ image processing pipeline (QPTiff) viewed using Phenochart ™ showing saturated signal from markers CD31, SCA1, and Endomucin (all red), CD41 (blue) and α-SMA (green). (B) Output image using our image processing described in our protocol of the same tissue showing specific and clear labeling of CD31, SCA1, and Endomucin (all red), CD41 (blue) and α-SMA (green).

## Slide 3
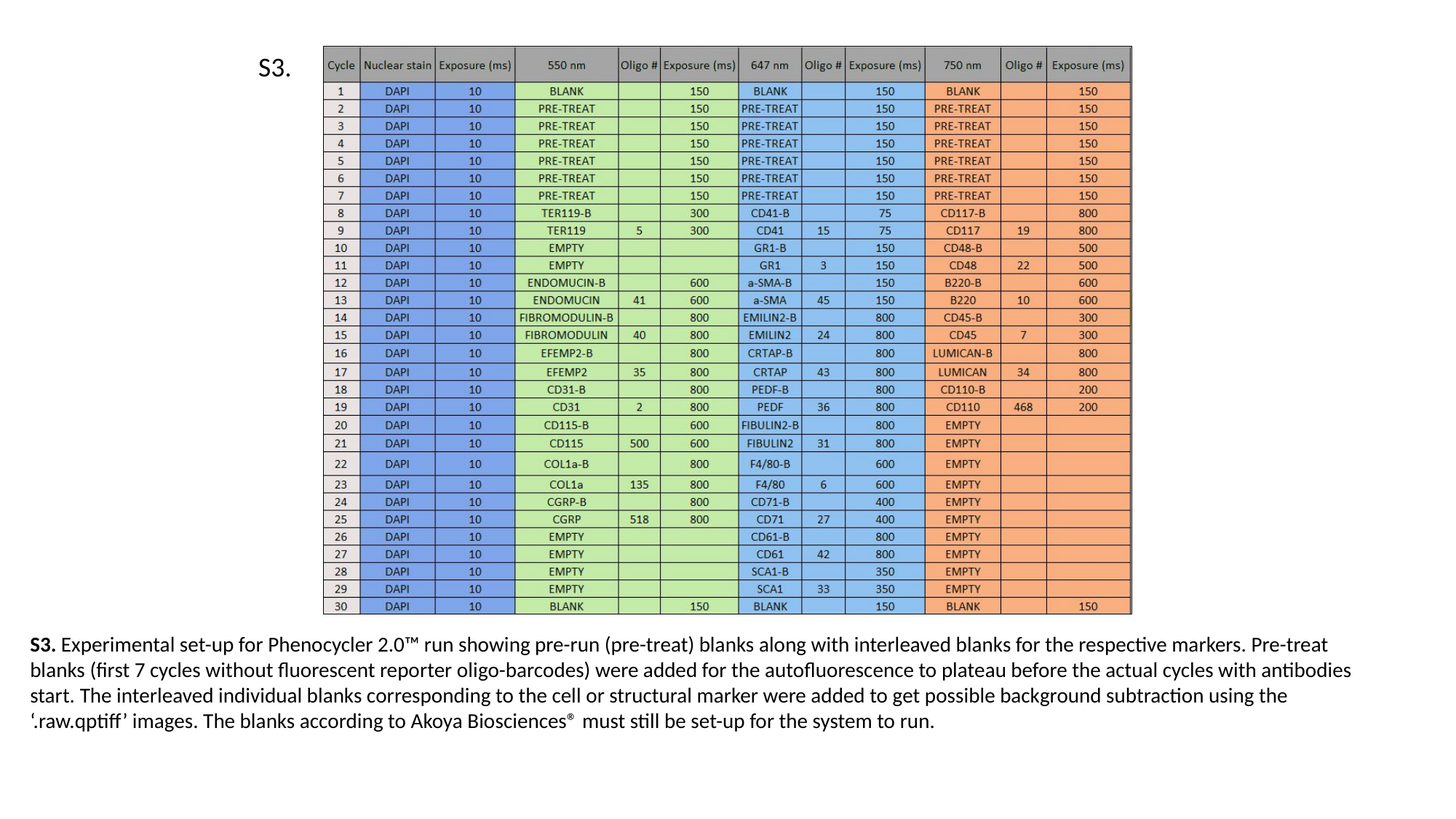

S3.
S3. Experimental set-up for Phenocycler 2.0™ run showing pre-run (pre-treat) blanks along with interleaved blanks for the respective markers. Pre-treat blanks (first 7 cycles without fluorescent reporter oligo-barcodes) were added for the autofluorescence to plateau before the actual cycles with antibodies start. The interleaved individual blanks corresponding to the cell or structural marker were added to get possible background subtraction using the ‘.raw.qptiff’ images. The blanks according to Akoya Biosciences® must still be set-up for the system to run.

## Slide 4
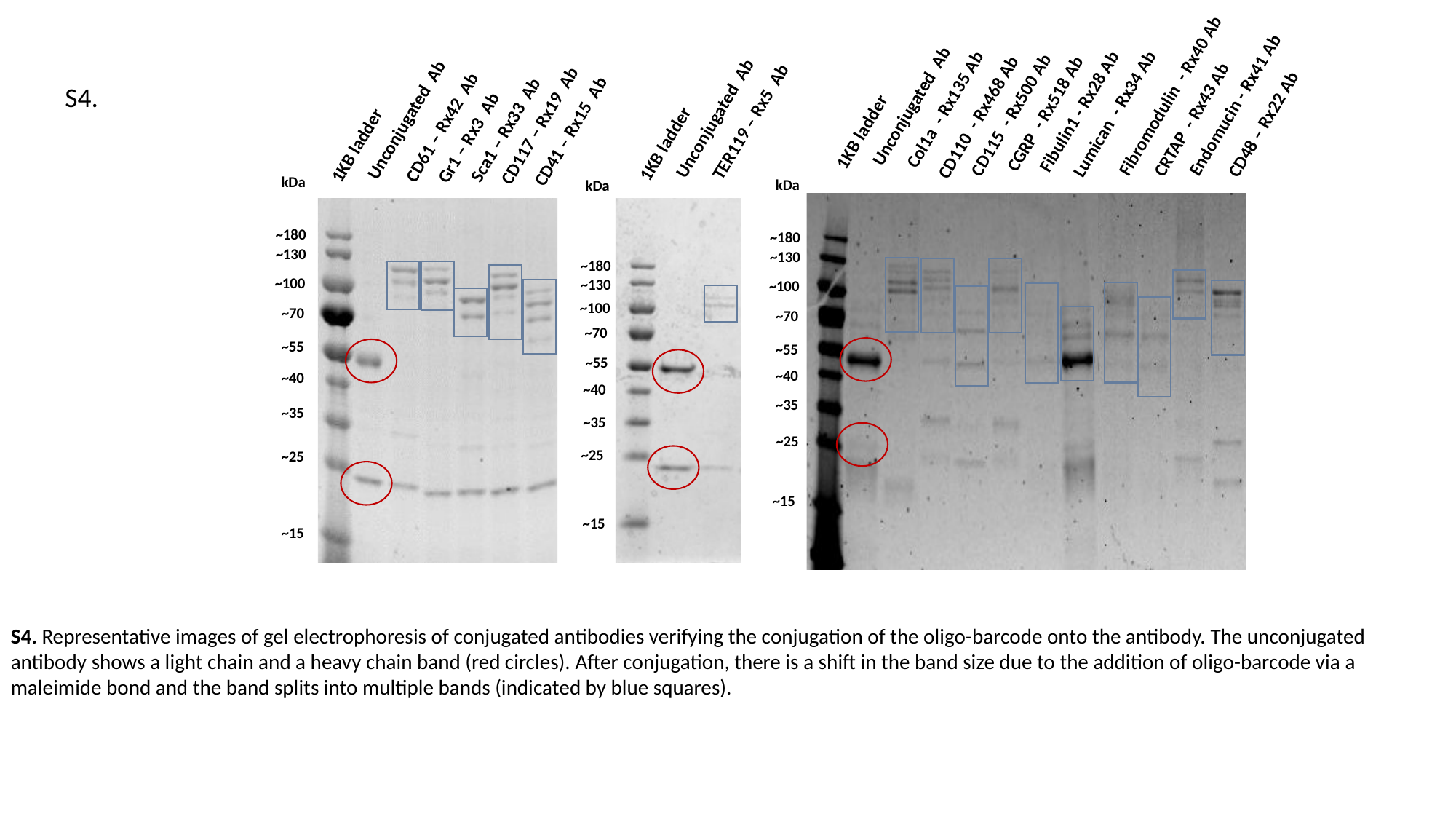

Fibromodulin - Rx40 Ab
Endomucin - Rx41 Ab
Unconjugated Ab
Col1a - Rx135 Ab
Fibulin1 - Rx28 Ab
Lumican - Rx34 Ab
CGRP - Rx518 Ab
CD115 - Rx500 Ab
CD110 - Rx468 Ab
CRTAP - Rx43 Ab
CD48 – Rx22 Ab
1KB ladder
Unconjugated Ab
TER119 – Rx5 Ab
1KB ladder
Unconjugated Ab
CD117 – Rx19 Ab
CD61 – Rx42 Ab
Sca1 – Rx33 Ab
CD41 – Rx15 Ab
Gr1 – Rx3 Ab
1KB ladder
S4.
kDa
kDa
kDa
~180
~180
~130
~130
~180
~100
~130
~100
~100
~70
~70
~70
~55
~55
~55
~40
~40
~40
~35
~35
~35
~25
~25
~25
~15
~15
~15
S4. Representative images of gel electrophoresis of conjugated antibodies verifying the conjugation of the oligo-barcode onto the antibody. The unconjugated antibody shows a light chain and a heavy chain band (red circles). After conjugation, there is a shift in the band size due to the addition of oligo-barcode via a maleimide bond and the band splits into multiple bands (indicated by blue squares).

## Slide 5
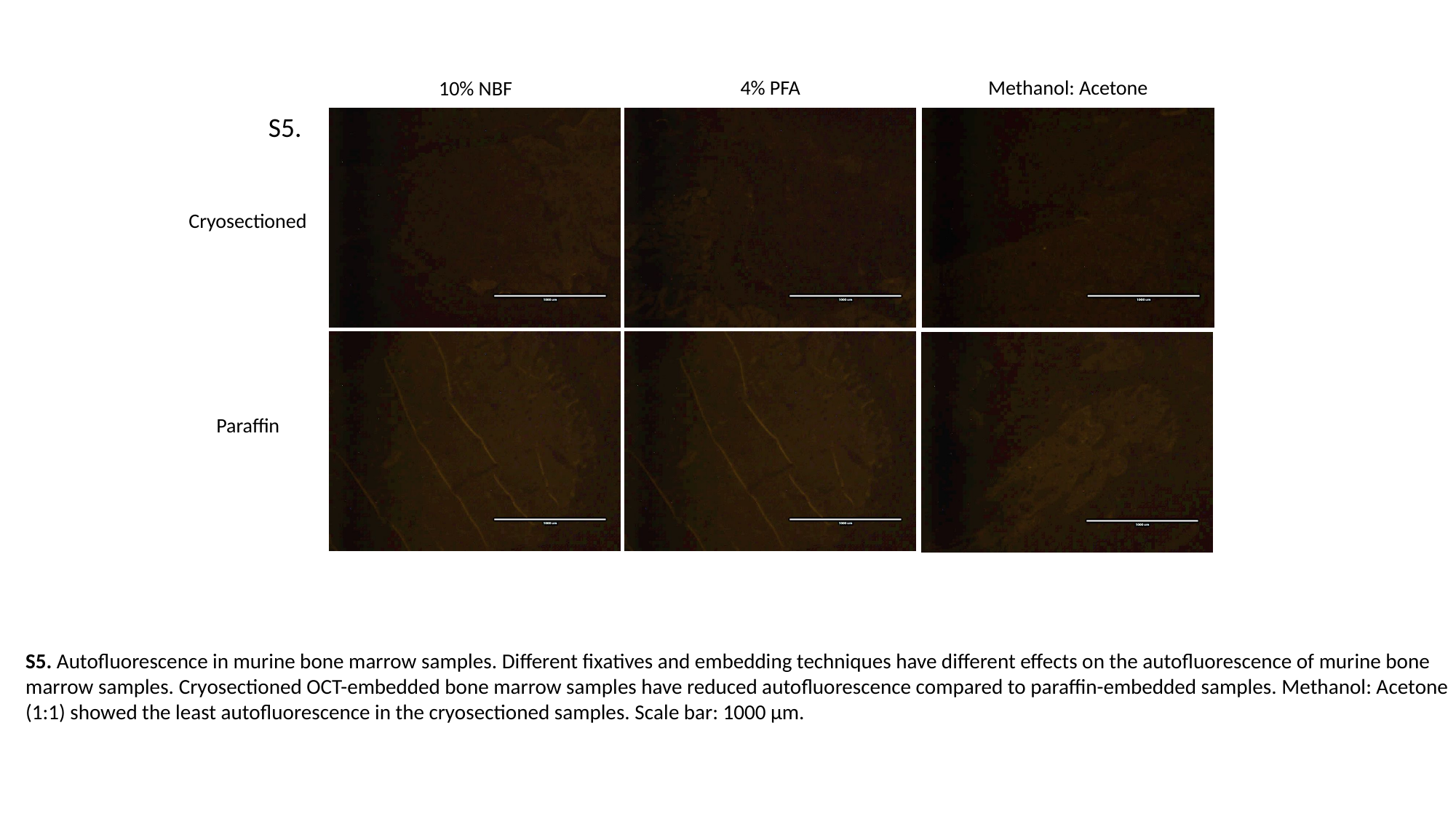

4% PFA
Methanol: Acetone
10% NBF
Cryosectioned
Paraffin
S5.
S5. Autofluorescence in murine bone marrow samples. Different fixatives and embedding techniques have different effects on the autofluorescence of murine bone marrow samples. Cryosectioned OCT-embedded bone marrow samples have reduced autofluorescence compared to paraffin-embedded samples. Methanol: Acetone (1:1) showed the least autofluorescence in the cryosectioned samples. Scale bar: 1000 µm.

## Slide 6
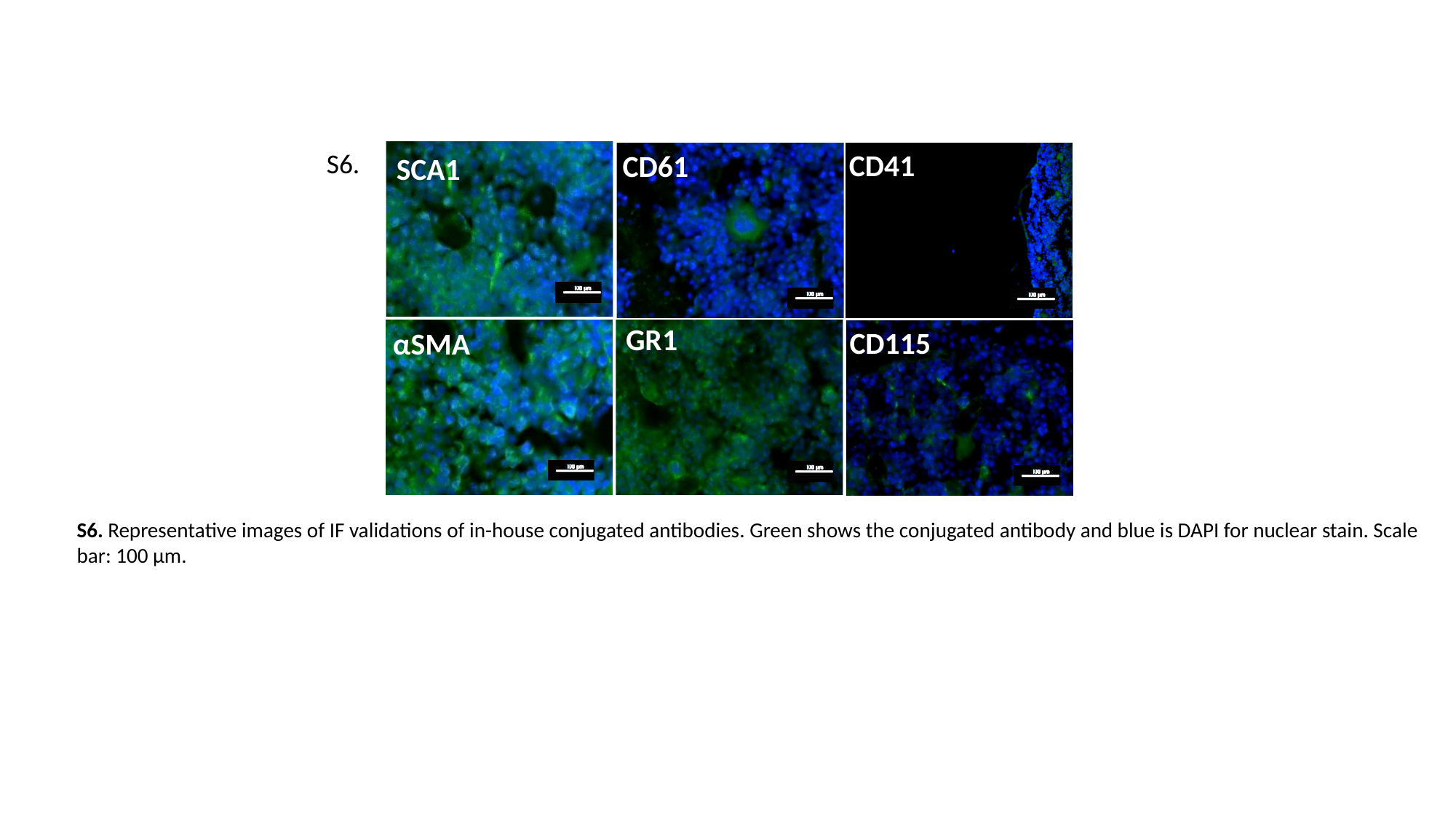

S6.
CD41
CD61
SCA1
GR1
CD115
αSMA
S6. Representative images of IF validations of in-house conjugated antibodies. Green shows the conjugated antibody and blue is DAPI for nuclear stain. Scale bar: 100 µm.

## Slide 7
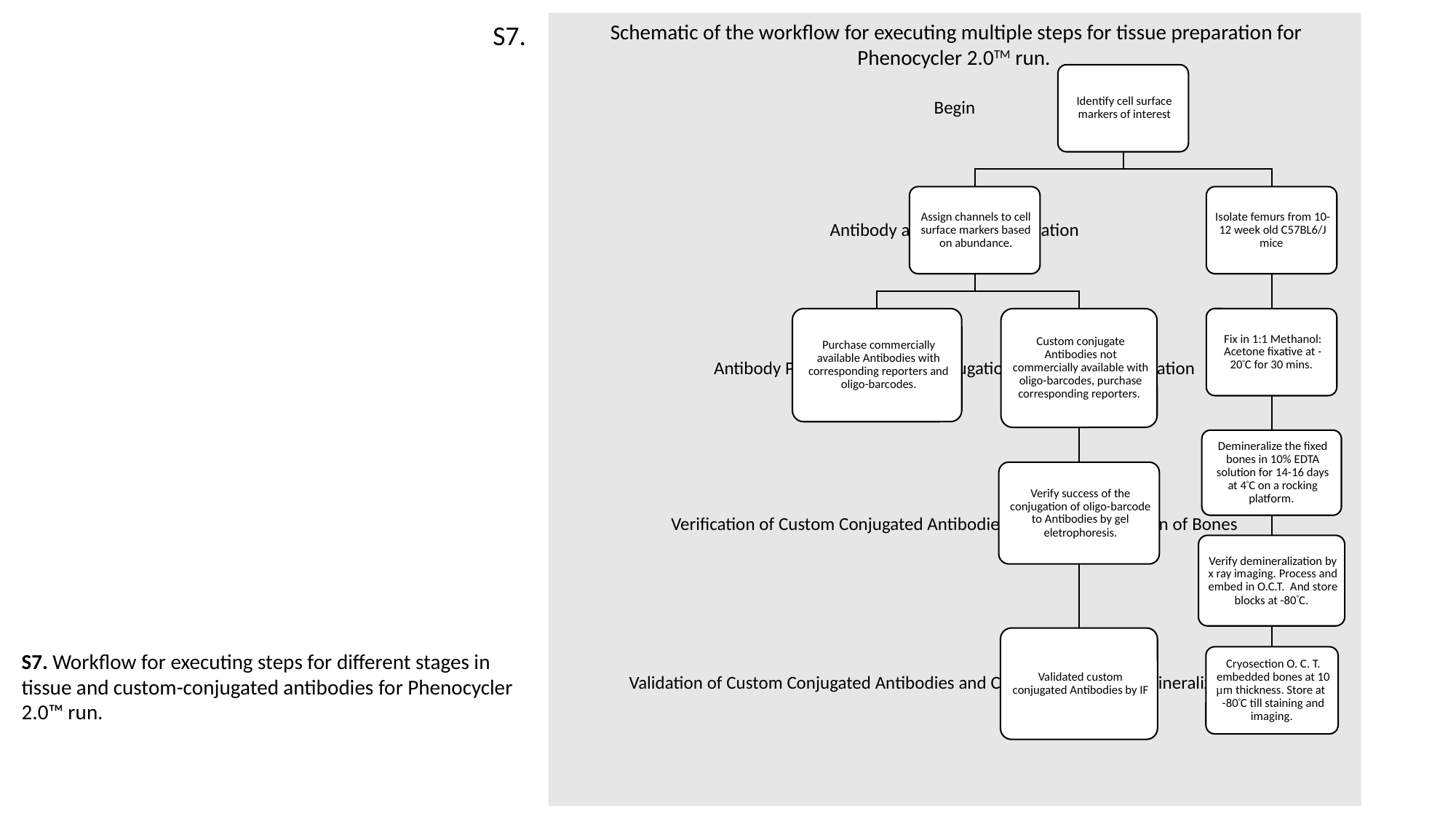

S7.
Schematic of the workflow for executing multiple steps for tissue preparation for Phenocycler 2.0TM run.
S7. Workflow for executing steps for different stages in tissue and custom-conjugated antibodies for Phenocycler 2.0™ run.

## Slide 8
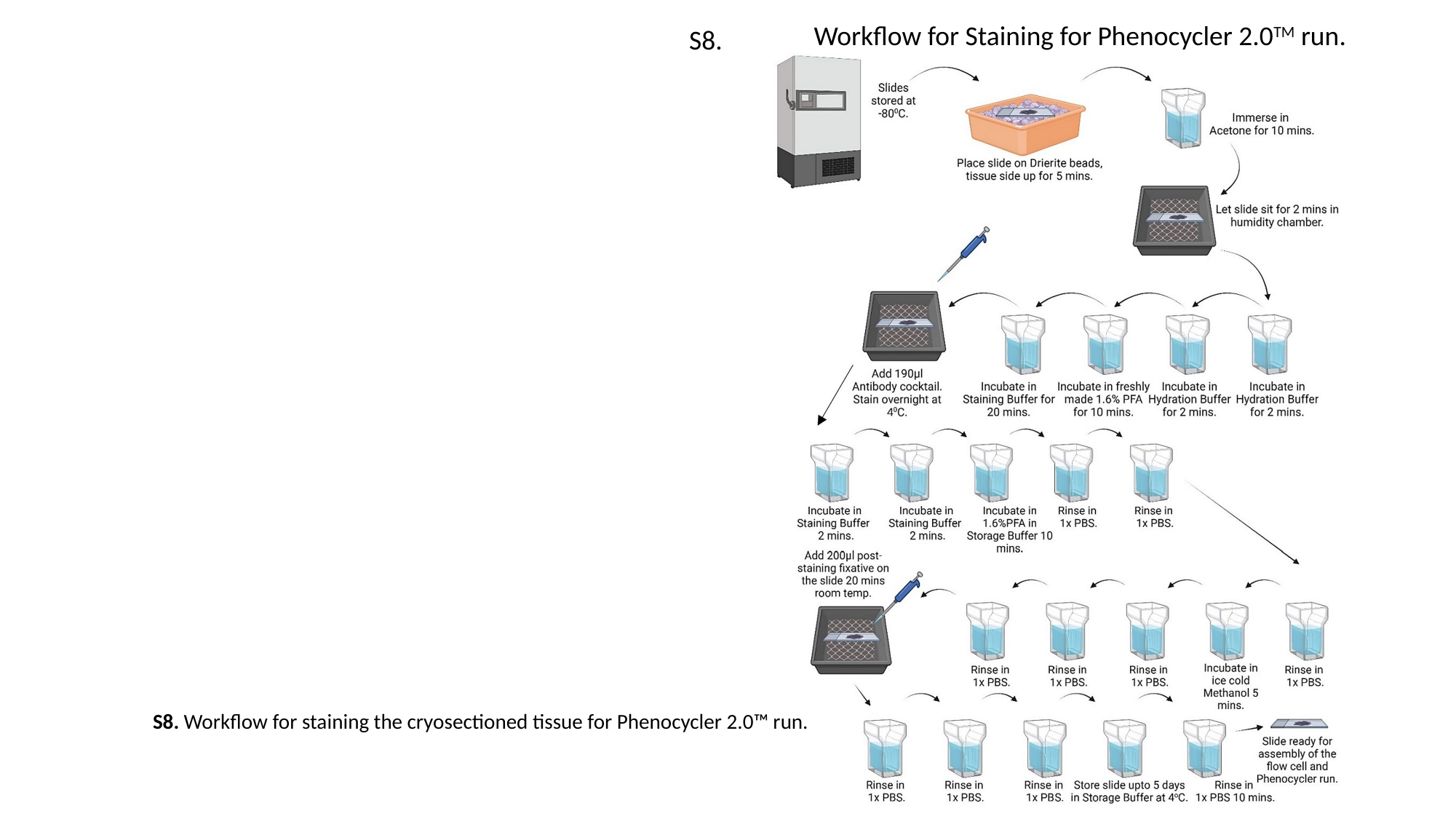

Workflow for Staining for Phenocycler 2.0TM run.
S8.
S8. Workflow for staining the cryosectioned tissue for Phenocycler 2.0™ run.
